# Distinct Molecular Strategies for Hox-Mediated Limb Suppression in *Drosophila*: From Cooperativity to Dispensability/Antagonism in TALE Partnership

**DOI:** 10.1371/journal.pgen.1003307

**Published:** 2013-03-07

**Authors:** Nagraj Sambrani, Bruno Hudry, Corinne Maurel-Zaffran, Amel Zouaz, Rakesh Mishra, Samir Merabet, Yacine Graba

**Affiliations:** 1 Centre National de la Recherche Scientifique (CNRS), Aix Marseille Université (AMU), Institut de Biologie du Développement de Marseille Luminy (IBDML, UMR 7288), Parc Scientifique de Luminy, Marseille, France; 2 Centre for Cellular and Molecular Biology (CCMB), Hyderabad, India; University of California San Francisco, United States of America

## Abstract

The emergence following gene duplication of a large repertoire of Hox paralogue proteins underlies the importance taken by Hox proteins in controlling animal body plans in development and evolution. Sequence divergence of paralogous proteins accounts for functional specialization, promoting axial morphological diversification in bilaterian animals. Yet functionally specialized paralogous Hox proteins also continue performing ancient common functions. In this study, we investigate how highly divergent Hox proteins perform an identical function. This was achieved by comparing in *Drosophila* the mode of limb suppression by the central (Ultrabithorax and AbdominalA) and posterior class (AbdominalB) Hox proteins. Results highlight that Hox-mediated limb suppression relies on distinct modes of DNA binding and a distinct use of TALE cofactors. Control of common functions by divergent Hox proteins, at least in the case studied, relies on evolving novel molecular properties. Thus, changes in protein sequences not only provide the driving force for functional specialization of Hox paralogue proteins, but also provide means to perform common ancient functions in distinct ways.

## Author Summary

Animal body plan diversity is controlled by transcription factors that select within each cell of a multi-cellular organism the set of genes to be expressed, eventually allowing distinct fate to emerge according to spatial coordinates. Transcription factors can be grouped based on their DNA binding domains in a few classes that likely arise from a common ancestral protein. This raises the question of how, within each class, transcription factors have gained specific function, and while doing so how they still continue performing ancient functions. Hox proteins, which play key roles in diversifying animal morphology, have largely been used to unravel the mechanisms underlying functional diversification of transcription factors. Here we use this family of transcription factors to investigate how common functions are achieved by divergent transcription factors. Results suggest that changes in protein sequences not only provide the driving force for defining novel and specific functions, but also provide means to perform common ancient functions in distinct ways.

## Introduction

Hox genes encode homeodomain (HD) containing transcription factors widely used for diversifying animal body plans in development and evolution [1]–[3]. The Hox gene repertoire most likely arose from tandem duplication events of ancestral genes, followed by sequence divergence that promoted the emergence of up to 14 paralogous groups in vertebrates [4]. The emergence of a large repertoire of Hox proteins certainly underlies the importance the Hox gene family has acquired in promoting morphological diversification of most animal body parts in higher eukaryotes.

Sequence conservation/divergence within the HD allows grouping paralogue proteins in three classes [5]–[8]. These classes correlate with the A–P deployment of Hox gene expression patterns as well as with the location within Hox clusters, and were accordingly termed anterior, central and posterior. Anterior class Hox genes (Hox1-3) are expressed most anteriorly and are located 3′ in the Hox clusters; central class Hox genes (Hox4-8) are expressed in medial region of the embryo and are located centrally in the clusters; posterior class Hox genes (Hox9-13) are expressed most posteriorly and are located most 5′ in the clusters. The sequence divergence of Hox proteins, including within the HD that constitutes the unique DNA binding domain of the Hox transcription factors, allows Hox paralogue proteins to display distinct regulatory functions, promoting axial morphological diversification in all bilaterian animals [3], [5], [9], [10].

Yet, in addition to having specialized biological functions, distinct Hox paralogue proteins also perform common (identical) functions. A striking example is provided by the functional equivalence of most *Drosophila* Hox paralogue proteins in specifying tritocerebral commissure in the embryonic brain [11]. Such common biological functions may represent remanent functions already assumed by the Hox gene from which the paralogue genes originate, which may then rely on ancestral properties still present in the divergent paralogue proteins. Alternatively common functions may rely on evolving novel properties.

We aimed at addressing this so far poorly investigated issue by comparing in *Drosophila* the mode of action of central and posterior class Hox proteins, which display the most extreme divergence within Hox paralogues [4]. Ultrabithorax (Ubx) and AbdominalA (AbdA), two central class Hox proteins, were proposed to arise from a recent gene duplication, have highly conserved HDs (8% of divergence within the HDs) and share additional protein domains, including the Hexapeptide (HX) motif upstream of the HD, as well as a short peptide downstream of the HD, termed UbdA [3]. Although not limited to this function, both motifs have been shown to promote the recruitment of the PBC class cofactor Extradenticle (Exd) [12]–[15]. In contrast, AbdB that arose from a more ancient duplication has a HD that largely diverges from that of Ubx and AbdA (41% of divergence within the HDs). In addition AbdB lacks the Ubx/AbdA specific UbdA domain, and lack a canonical HX motif, although a key Exd interacting residue within this domain remains conserved [16].

To assess molecularly how divergent Hox proteins as Ubx/AbdA and AbdB can perform identical functions, we focused on limb suppression. As all insects, *Drosophila* harbors limbs exclusively in the thorax and not in the abdomen. This morphological distinction relies on the regulation of the limb-promoting gene *Distalless* (*Dll*), expressed in the thoracic limb primordia, but not in the abdomen. Thoracic specific expression of *Dll* relies on abdominal repression by Ubx and AbdA in the anterior abdomen (segments A1-A7) and by AbdB in the posterior abdomen (segments A8-9) [17]. Localized thoracic *Dll* expression was shown to be mediated by multiple enhancers. This includes two enhancers in the 5′ and a distant one in the 3′ of the gene [17], [18]. Each of this enhancer displays distinct temporal and spatial specificities, which likely contribute to the developmental dynamic expression pattern in the leg primordia. One of the 5′ enhancer, Dll304, has been extensively analyzed, leading to a good molecular understanding of *Dll* repression by Ubx and AbdA [13]–[15], [19]–[21]. The Ubx/AbdA-mediated transcriptional repression is mediated within a 57-base-pair (bp) repressor element (DMX-R). This element harbors functional binding sites for Ubx/AbdA proteins, for two TALE proteins (a special class of HD containing proteins with a Three Amino acid inserted in between Helix 1and 2), the PBC class cofactors Extradenticle (Exd) and the Meis/Prep class cofactor Homothorax (Hth), and for the compartment specific proteins Engrailed (En) and Sloppy paired (Slp). As is the case for the regulation of other Hox target genes, Exd and Hth were shown to cooperatively bind DNA with Ubx and AbdA, while En and Slp, which both harbor a Groucho interacting domain, may in turn recruit a Groucho containing corepressor complex. In this study, we dissected the molecular modalities underlying AbdB-mediated repression of *Dll*, which allows addressing how posterior and central class Hox proteins perform similar functions.

## Results

### AbdB represses *Dll* in abdominal segments A8 and A9

**Figure 1:**
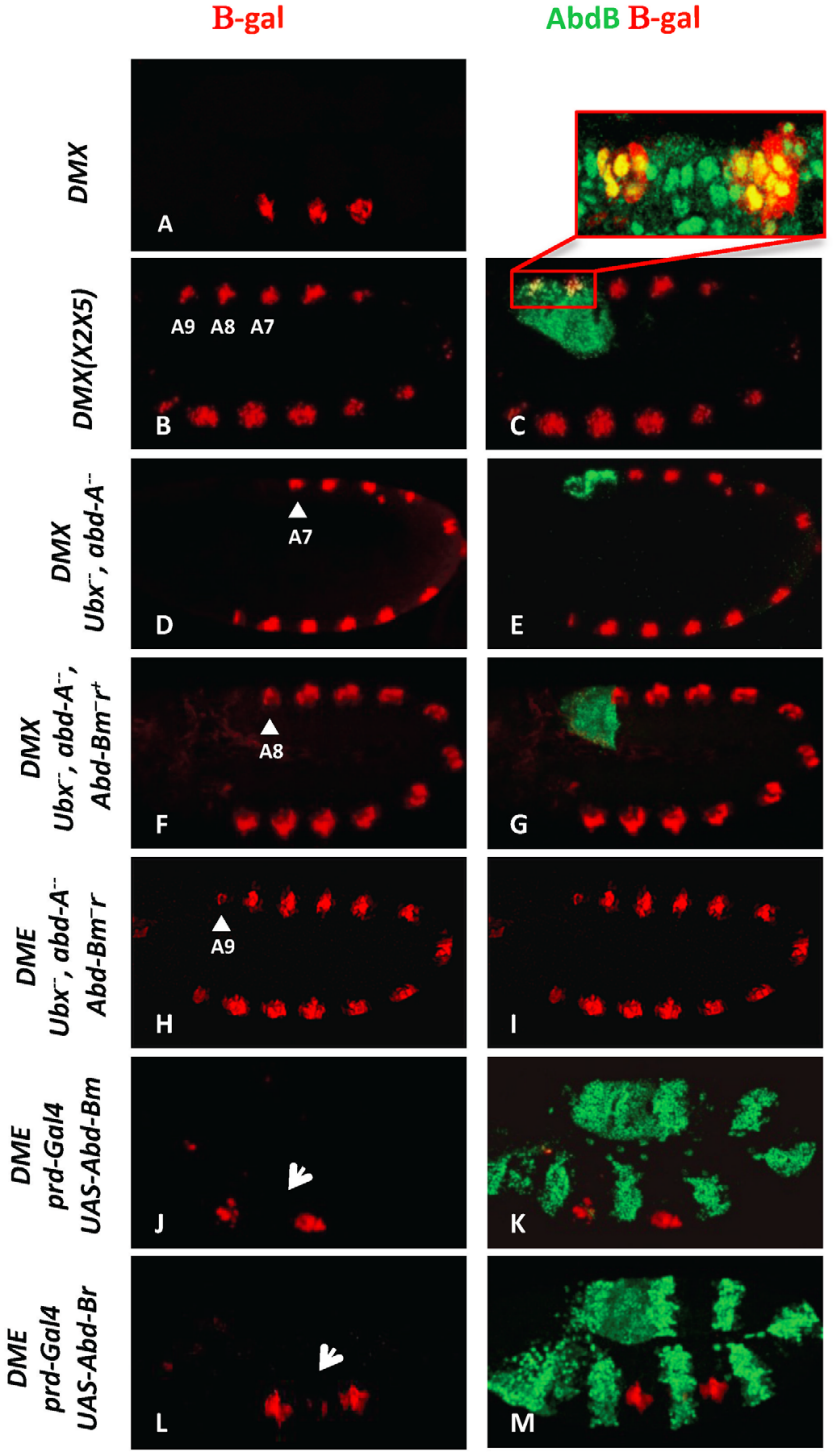
AbdB m and r isoforms repress *Dll* in the posterior abdominal segments A8 and A9. (A) Embryo stained for β-gal driven by the DMX enhancer (red) showing restricted thoracic activity. (B–C) DMX(X2X5) embryos co-stained for β-gal (red) and AbdB (green). This modified DMX enhancer is derepressed in all abdominal segments, including A8 and A9. Co-localization of β-gal and AbdB in A8 and A9 cells normally subject to enhancer activity repression is highlighted in the magnified view. (D–E) Embryo lacking Ubx and AbdA function (*Df(109)*) shows DMX abdominal de-repression (red) up to A7 (arrowhead) and remains repressed in A8 and A9 segments where AbdB is expressed at high levels (green). (F–G) Embryo lacking Ubx, AbdA and the AbdBm isoform, but retaining the AbdB r isoform, shows derepression of DMX (red) till A8 (arrowhead). (H–I) Embryos lacking Ubx, AbdA and the AbdB m and r isoforms (*Df P9*) show DMX de-repression (red) in all abdominal segments, including in A9 (arrowhead). (J–K) *prd-Gal4* driven ectopic expression of the AbdBm isoform represses DMX activity (arrow in T2). (L–M) *prd-Gal4* driven ectopic expression of the AbdBr isoform represses DMX activity (arrow in T2).

Loss and gain of function data supports a role of AbdB in repressing *Dll* expression (Figure S1; [17]). To explore further the mechanism of AbdB mediated *Dll* repression, we first asked if AbdB is present in cells with the potential to express *Dll*. *Dll* expression and regulation was followed using *Dll* reporter genes, DMX or DME (when the experiments involved the paired(prd)-Gal4 driver, see material and methods), that both accurately reproduce *Dll* expression (Figure 1A) and that only differs in the 3′ sequence by a few nucleotides that provide DMX with a second Hox binding site [20]). We first took advantage of the DMX(X2X5) that bears mutations in binding sites for the En (X5) and Slp (X2) proteins [20]. DMX(X2X5) drives *lacZ* reporter expression in the thorax, as wild type DMX (Figure 1A), but also in the abdomen, including segments A8 and A9 (Figure 1B). Co-staining with AbdB antibodies showed that cells normally repressing DMX in A8 and A9, identified by DMX(X2X5) activity, accumulate AbdB (Figure 1C).

The *AbdB* gene produces two isoforms: AbdBm in segments A8 (also expressed in A5–A7 albeit at lower levels) and AbdBr in segment A9 [22]. In the absence of Ubx and AbdA proteins but in the presence of an intact *AbdB* gene, DMX activity is de-repressed in abdominal segments A1–A7, but not in A8 and A9 (Figure 1D, 1E). Removing in addition the AbdBm isoform results in expanding the derepression of DMX to A8 (Figure 1F, 1G), while further deleting the AbdBr isoform results in full abdominal derepression, including A9 (Figure 1H, 1I). Taken together, these results indicate that the AbdBm and AbdBr isoforms are responsible for DMX repression in A8 and A9 segments respectively. The repressive activity of AbdB isoforms was further investigated in gain of function experiments. AbdB isoforms were ectopically expressed in every other segments with the paired (prd)-Gal4 driver [19]. Results indicate that both isoforms are equally efficient in repression (Figure 1J–1M), further validating repression by AbdB m and r isoforms. To investigate in more depth the repression of *Dll* by AbdB we focused on the AbdBm isoform that for simplicity will be referred to as AbdB in the remaining text.

### AbdB-mediated repression of *Dll* requires the compartment specific cofactors En and Slp

**Figure 2:**
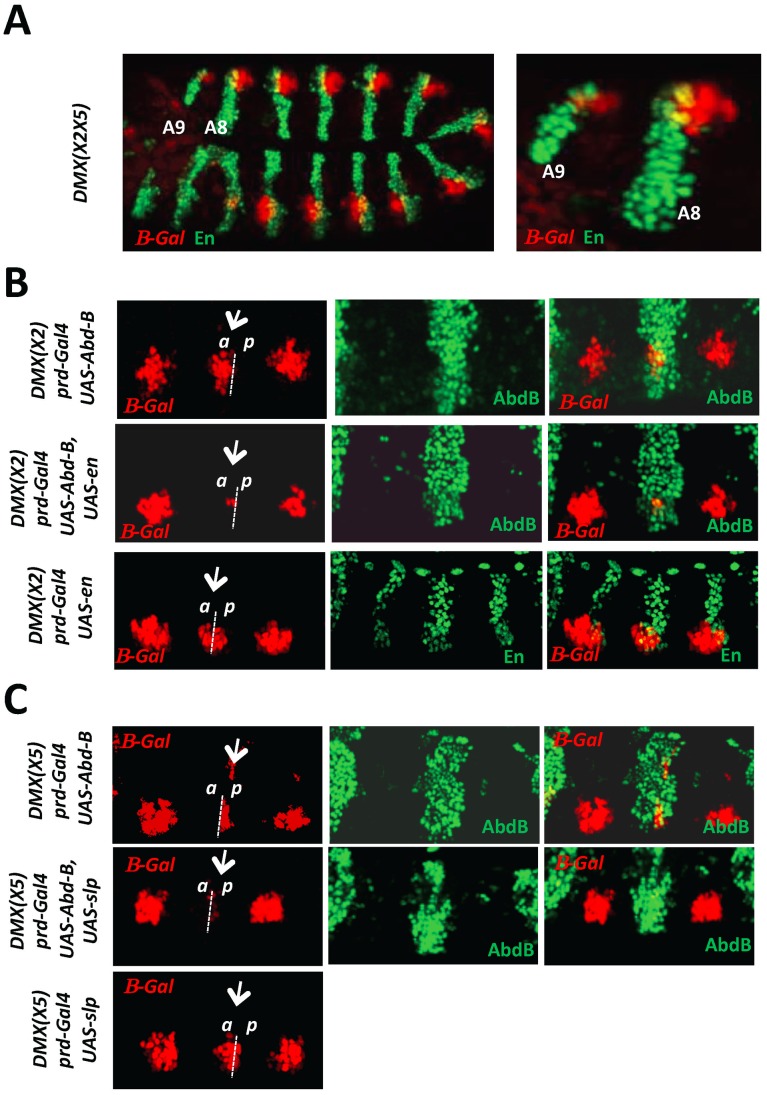
AbdB-mediated repression of DMX requires the activity of posterior and anterior compartment specific cofactors En and Slp. A) Embryo co-stained for β-gal driven by DMX(X2X5) (red) and En (green). Magnification of the posterior abdominal segments A8 and A9 highlights derepression of DMX(X2X5) in anterior (En negative) and posterior (En positive) compartment cells. B) Thoracic segments of embryos bearing the DMX(X2) reporter and expressing ectopically AbdB, En or AbdB and En, in every other segments driven by the prd-GAL4 driver. The repressive potential on DMX(X2) is evaluated in the thoracic T2 segment (arrow). Upper panels: In embryos ectopically expressing AbdB, repression only occurs in posterior compartment cells (p). Middle panels: In embryos ectopically expressing AbdB and En, repression occurs in both posterior (p) and anterior compartment cells (a). Lower panels: In embryos ectopically expressing En, only weak and compartment non-specific repression is observed. C) Thoracic segments of embryo bearing the DMX(X5) reporter and expressing ectopically AbdB, Slp or AbdB and Slp, in every other segments driven by the prd-GAL4 driver. The repressive potential on DMX(X5) is evaluated in the thoracic T2 segment (arrow). Upper panels: In embryos ectopically expressing AbdB, repression occurs in anterior compartment cells (a). Middle panels: In embryos ectopically expressing AbdB and Slp, repression occurs in both anterior (a) as well as in posterior compartment cells (p). Lower panels: In embryos ectopically expressing Slp, only weak and compartment non specific repression is observed.

Repression of DMX by Ubx and AbdA was shown to rely on the compartment specific proteins En and Slp. We first asked whether de-repression in A8 and A9 segments occurs both in anterior and posterior compartment cells. This was achieved by following the distribution of En, that identifies posterior compartment cells, and LacZ driven by the DMX(X2X5), in the posterior abdomen. Results unambiguously show that as in the anterior abdomen, derepression in A8-9 occurs both in En negative and positive cells (Figure 2A), indicating that AbdB-mediated repression occurs both in anterior and posterior compartments.

Next we investigated the contribution of En and Slp proteins for AbdB-mediated DMX repression. The requirement of En and Slp for proper *Dll* activation in thoracic segments precludes a loss of function approach. The question was addressed in gain of function experiments, making use of DMX enhancers mutated either on the Slp or on the En binding sites [20]. Regarding the contribution of En to AbdB-mediated repression, the rational behind the experiment was to assay the role of En in anterior compartment cells. Upon mutation of the Slp binding site (DMX(X2)), expression of AbdB in T2 using the prd-Gal4 driver represses DMX(X2) exclusively in posterior compartment cells. This repression uses the endogenous En protein and the intact En binding site within DMX(X2) (Figure 2B, upper panel). In contrast, repression in anterior compartment cells does not occur as the endogenous Slp protein can not bind DMX(X2). However, if En is crucial for AbdB-mediated DMX repression, the lack of repression in these anterior compartment cells should be compensated if En is provided in anterior compartment cells, as repression then could occur by use of the En cofactor, for which the binding site in DMX(X2) is not mutated. Co-expression of AbdB and En in T2 results in repression of DMX(X2) both in anterior and posterior compartment cells (Figure 2B, middle panel). No repression was observed when En is expressed in the absence of AbdB (Figure 2B, lower panel). Taken together these experiments provide functional support for a role of En in AbdB-mediated DMX repression. Through a similar strategy, using DMX(X5) mutated in the En binding site, and comparing the repressive effect of AbdB in the presence or absence of Slp in posterior compartment cells, we also establish a requirement of Slp for AbdB-mediated DMX repression (Figure 2C).

We concluded, as previously shown for Ubx/AbdA, that AbdB-mediated repression of DMX occurs in anterior and posterior compartment cells and uses the En and Slp co-repressors.

### Dispensability/antagonism in AbdB TALE partnership

**Figure 3:**
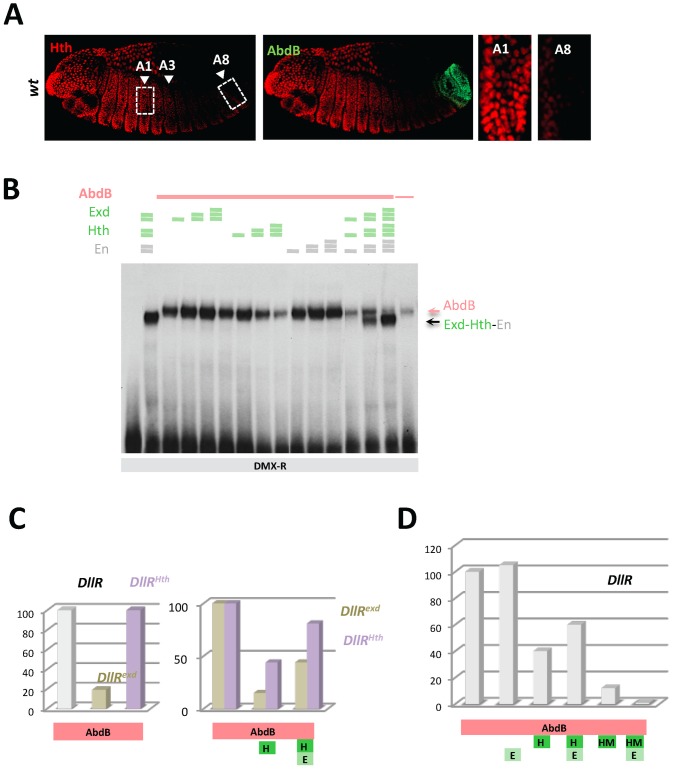
Dispensability of Exd/Hth cofactors for AbdB-mediated DMX posterior repression in posterior segments. A) Embryos co-stained for Hth (red) and AbdB (green). Arrowheads point to segments A1, A3 and A8. wild type embryo, decrease of Hth expression is seen from segment A3 reaching almost undetectable levels in A8. B) EMSA of DMX-R (containing binding sites Slp,Hox1, Exd, En, Hth and Hox2) with AbdB and increasing amounts of Exd, Hth, En, or En and with combined increasing amounts of Exd, Hth and En. The amount of AbdB remains constant whenever present, except in the last lane (depicted by a thin pink line) where 1/3 of this quantity was used. Exd-Hth-En/DNA and AbdB/DNA complexes are highlighted by arrows. C) Quantification of AbdB binding in EMSA to DIIR (containing binding sites Hox1, Exd, En, and Hth) mutated in the Exd (DIIR*^exd^*) or Hth (DIIR*^Hth^*) binding sites in the presence of AbdB alone, with Exd or Exd and Hth (see Figure S4). Note that mutation of the Exd binding site affect the formation of AbdB/DNA complexes. For the ease of comparison, AbdB binding to DIIR*^exd^* and DIIR*^Hth^* have been arbitrarily set to 100%, allowing assessing the effect of Hth and Hth/Exd inhibitory effects independently off the effect of binding site mutations on AbdB/DNA complex assembly. D) Quantification of AbdB binding in EMSA to DIIR with various combinations of AbdB, Exd, Hth and truncated HM (HD less) form of Hth (See Figure S4). Note that the Exd-mediated release of inhibitory effect seen for full length Hth is lost with the truncated Hth HM protein.

AbdA and Ubx efficiently bind DMX-R only in the presence of Exd and Hth, and binding sites for these two TALE proteins are required for efficient repression by Ubx and AbdA [19], [20]. To address the contribution of Exd/Hth to AbdB-mediated *Dll* repression, we first examined the distribution of Exd. Consistent with previous reports [23], we found that while being expressed at high levels in the thorax and anterior abdomen, nuclear protein accumulation decreases starting from segment A3, with no or barely detectable levels present in A8 and A9, where AbdB is expressed at high levels and represses *Dll* (Figure S2). We also examined the expression of Hth, and found that it follows Exd protein accumulation, consistent with its known function in promoting nuclear accumulation of Exd (Figure 3A). These observations indicate that unlike Ubx and AbdA, AbdB may not require the TALE cofactors Exd and Hth for binding DMX-R and repressing *Dll*.

The requirement of Exd and Hth for AbdB binding to DMX-R element was investigated by EMSA. Results showed that AbdB binds efficiently *Dll* cis sequences in the absence of Exd and Hth (Figure 3B), and that full AbdB binding requires the integrity of the Hox1 and Hox2 binding sites, but also that of the “Exd” binding site (Figure S3A, S3B). Addition of Exd, Hth as well as En either separately or in combination does not improve AbdB binding, and does not allow the assembling of an AbdB-Exd-Hth (or AbdB-Exd-Hth-En) on *Dll* sequences (Figure 3B and Figure S3C). It was rather found that the presence of the Hth protein, either alone or within a trimeric Exd-Hth-En complex, inhibits AbdB monomer binding (Figure 3B). These results reveals a Hox/TALE partnership distinct from that seen for Ubx and AbdA, with Exd, En and Hth being dispensable for AbdB binding, and Hth and Hth-containing complexes (Hth-Exd and Exd-Hth-En) providing an inhibitory effect on AbdB binding (similar results are shown in [24]).

To further investigate the molecular bases of this competitive partnership, we first investigated the DNA binding requirement of Hth and Exd for proper competitive effect. Results showed that mutation of the Hth or Exd binding sites do not impair the Hth-mediated inhibition of AbdB binding. Normalizing AbdB binding with reference to its binding to the mutated *DII* probes in absence of Hth further showed the efficiency of AbdB binding is not weaker than that observed with wild type probe (Figure 3C and Figure S4), indicating that AbdB binding inhibition by Hth does not require the Hth or Exd binding sites. This was further confirmed by the observation that a HD deleted form of Hth, Hth^HM^, that does not bind DNA, still efficiently inhibits AbdB binding (Figure 3D and Figure S4). Surprisingly however, the presence of Exd increases the inhibitory role of Hth^HM^, while it decreases that of full length Hth (Figure 3D and Figure S4). This could be explained by the assembling of a Exd-Hth-DNA complex only in the case of the HD containing Hth protein (Figure S4), which lowers the availability of free Hth for competing AbdB DNA binding.

Similar experiments were conducted by adding the Hth and Exd proteins, in order to assess the inhibitory role of the Hth-Exd complex (Figure 3C, 3D and Figure S4). Results showed that as for Hth alone, inhibition of AbdB binding by the Hth-Exd complex does not require the Exd and Hth binding sites, although in the case of the Hth mutated probe, inhibition is weaker than on the wild type probe. We concluded that Hth and Hth-Exd mediated inhibition of AbdB binding relies on a mechanisms that do not require Hth or Hth-Exd binding to DNA, indicating that AbdB-Hth (or AbdB-Hth-Exd) interaction occurring outside DNA prevents AbdB binding (similar results are reported in [24]). These results however do not exclude that Hth (and Exd-Hth) also inhibits AbdB DNA binding by competing for overlapping binding sites, as is the case for Exd.

### AbdB- and Ubx/AbdA-mediated repression of *Dll* relies on the same DMX cis sequences that are either identically or distinctly used

The repressive function of DMX was shown to rely on a 57 bp element, named DMX-R, which was scanned for mutations affecting its capacity to mediate repression [20]. Among the 23 scanning mutations (2 to 5 base pair substitution), 8 were shown to result in strong abdominal derepression, identifying binding sites for the Hox proteins Ubx and AbdA, for the Hox TALE cofactors Exd and Hth, as well as for the corepressors En and Slp. In addition, mutations in the distal part of DMX-R result in weak abdominal derepression, identifying a second Hox binding site (Hox2). This Hox2 binding site is dispensable for proper repressive activity as the DME element conveys full abdominal repression.

**Figure 4:**
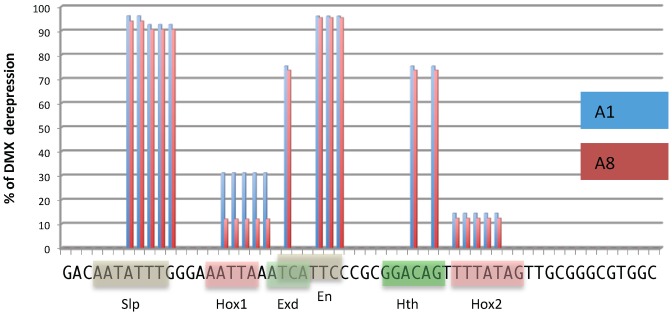
DMX-R cis sequence requirements for repression by Ubx/AbdA and AbdB. Schematic representation of cis sequence requirements for DMX repressive activity in A1 (Ubx/AbdA- mediated, blue) and A8 (AbdB-mediated, red) segments. 100% derepresion was defined by the level of abdominal DMX derepression in embryos fully deficient for Ubx, AbdA and AbdB m and r isoforms (*Df P9*). Cis sequence requirement was evaluated by quantifying the levels of derepression of 18 mutated forms of DMX-R (see Figures S5 and S6). These scanning mutations (altering simultaneously two to 5 nucleotide positions) cover 42 of the 57 nucleotide positions of the DMX-R element. Sequence is annotated according to transcription factor binding site (Slp, Exd, Hth, En and Hox) allocation from [20].

To see if the AbdB-mediated repression in A8-9 segments relies on the use of the same cis sequences as repression by Ubx and AbdA in the anterior abdomen (A1-7), we re-examined the effect associated to mutations spanning the DMX-R (19 of the 23 initial mutations) by exploring if any of these result in distinct effects in anterior abdominal segments, where repression is mediated by Ubx/AbdA, and posterior abdominal segments where repression is mediated by AbdB. This was achieved by quantifying the level of derepression associated to each mutation, focusing on segments A1 and A8, as representatives of anterior and posterior abdominal segments respectively. Results summarized in Figure 4 (see Figures S5, S6 for full data) show that no qualitative differences for cis requirements in A1 and A8 are seen: all positions of DMX-R not involved in repression in A1 are also not involved in repression in A8; all positions of DMX-R required for proper repression in A1 are also required for proper repression in A8. In one instance however, mutation of the Hox1 binding site, the level of derepression is distinct in A1 and A8, with a stronger derepression in A1 than A8. This quantitative distinction suggests that AbdB binds to additional cis sequences in DMX-R. In support of this, we found that mutation of the “Exd” binding site affects AbdB binding to DMX-R (see Figure 3C and Figure S3). We thus concluded that the same cis sequences in DMX-R are used for abdominal repression by AbdB in A8 and Ubx/AbdA in anterior abdominal segments. Yet this common requirement of cis sequences does not imply that these cis sequences are bound by the same proteins, as illustrated by the requirement of the “Exd” binding site for AbdB binding to DMX-R.

### AbdB represses Hth expression

**Figure 5:**
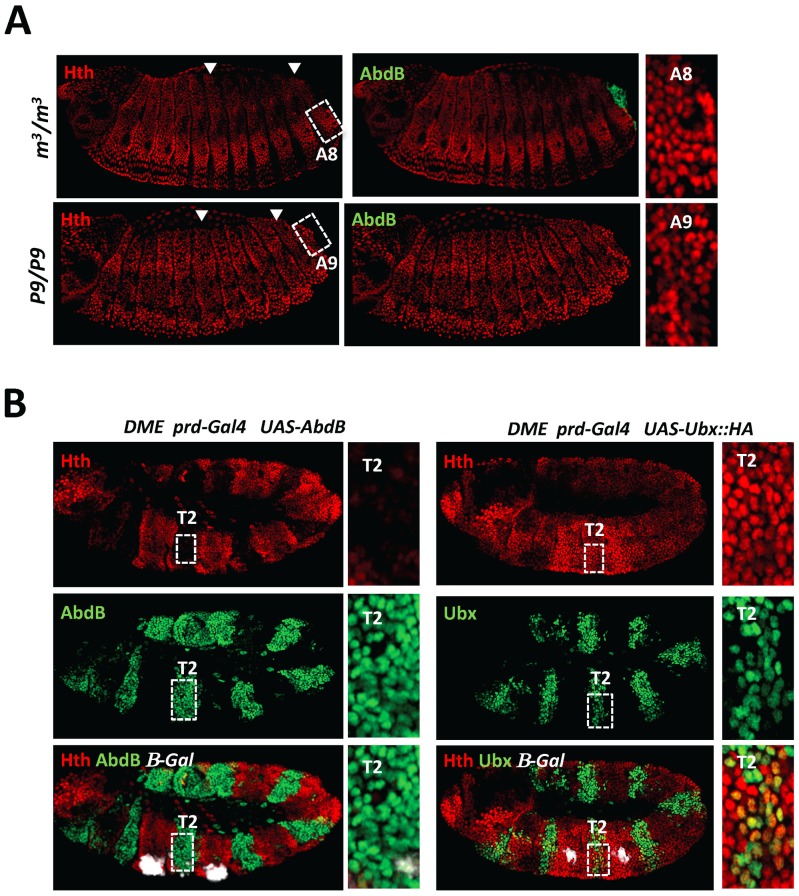
AbdB represses Hth expression. A) Embryos co-stained for Hth (red) and AbdB (green). Arrowheads highlight segments A3 and A8. Upper panels: embryo lacking AbdB m isoform (AbdB^m3^), displaying posterior derepression till A8 (compare to wild type embryo (Figure 3A)). Derepression does not spread to A9, where the AbdBr isoform is still expressed. Lower panels: embryo lacking both AbdB m and r isoforms show extension of Hth derepression to segment A9. Right panels are magnifications of A3 and A8/9 segments. B) Embryos bearing the DME reporter co-stained for β-gal (white), Hth (red) and AbdB (right panels, green) or Ubx (left panels, green). Ectopic AbdB expression was driven in every other segments by prd-Gal4. AbdB, but not Ubx ectopic expression, strongly represses Hth expression. Magnifications of thoracic T2 segments are shown.

The inhibitory role of Hth on AbdB binding to DMX-R suggests that down regulating the levels of Hth in the posterior abdomen is essential for proper AbdB-mediated *Dll* repression. Since Hth levels decrease dramatically in the posterior abdomen, we asked if AbdB itself mediates this down regulation. We found that depleting the AbdBm (*AbdB^m3^*) or AbdBm and r proteins (*Df(P9)*) results in increasing the level of Hth to a level similar to the anterior abdomen and thorax region, from segment A4 and including segments A8 and A9 respectively (Figure 5A). Although we do not visualize AbdB protein in segments as anterior as A4 where Exd and Hth start decreasing, AbdB transcripts are present till A4 and the functional domain of AbdB was delineated to segments A4–A9 [25], consistent with derepression of Hth in AbdB mutants starting from A4. The repressive role of AbdB on Hth expression was further confirmed in gain of function experiments, where it was found that AbdB has a strong repressive capacity on *hth* transcription (Figure S7) and Hth protein accumulation, when compared to Ubx (Figure 5B) or AbdA (Figure S8). Consistent with previous reports, similar conclusions could be reached for Exd nuclear accumulation in loss and gain of function experiments (Figure S2).

Finally, the importance of Hth downregulation for proper AbdB-mediated *Dll* repression was assessed by driving *hth* expression in the posterior abdomen, using the arm-Gal4 driver (Figure S9). In this condition however, we failed to efficiently induce high level of Hth protein accumulation in the posterior abdomen. This may suggest that low or absence of Hth protein accumulation in the posterior abdomen may be ensured by a double lock mechanism, one mediated by transcriptional repression, and the second one through a post-transcriptional mechanism, both potentially under AbdB control. Only a few embryos displayed a moderate level of Hth protein accumulation in the posterior abdomen and exhibited posterior derepression of DMX (Figure S9), indicating that absence of Hth is required for proper AbdB-mediated repression.

These data demonstrate that unlike Ubx and AbdA, AbdB binds DMX-R and represses *Dll* in cells where Hth and Exd have been dramatically down regulated, avoiding a competitive AbdB/Hth-Exd partnership.

### Intrinsic protein requirements for AbdB-mediated *Dll* repression: critical requirement of HD and HD C-terminal flanking residues

**Figure 6:**
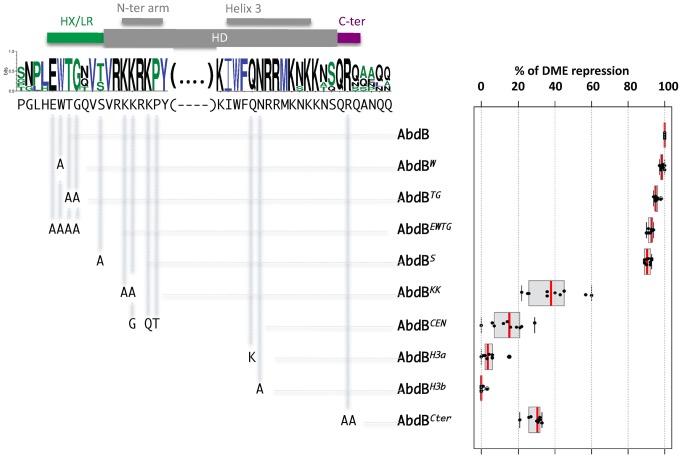
Protein sequence requirements for AbdB-mediated DME repression. Sequence conservation in the AbdB HD (shown only for the N-terminal arm and helix 3) and HD flanking regions HX/LR and C-ter region. Sequences upstream and downstream of these regions display progressively weaker conservation. Web logo was obtained using sequences the following AbdB sequences (Drosophila (AAA84402), Tribolium (AAF36721.1), Anophela (XM311628), Sacculine (AAQ49317.1), Folsomia (AAK52499.1) and human (BCO10023)). The *Drosophila melanogaster* sequence is shown below the web logo. The position and the nature of the mutations generated are represented below the web logo. Effects of the mutations on the repressive activity of AbdB on DME are displayed in a box plot representation. While mutations in the HX/LR region have little effect on AbdB repressive activity, mutations within the HD, including the N-terminal arm, helix 3 and the Cter alter to different extent AbdB repressive potential. Illustration of data is given in Figure S10.

To further examine the mode of AbdB-mediated *Dll* repression, we aimed at identifying residues of AbdB that would be critical for its repressive function. Sequence alignment of arthropod AbdB proteins revealed sequence conservation immediately adjacent to the HD (Figure 6). This includes a stretch of amino-acids flanking the HD N-terminally (EWTGQVS), with the W possibly representing a residual degenerated HX motif, which has only retained the core residue required for PBC class protein interaction [16], [26]–[28]. While dispensable for Ubx-mediated repression, the HX was shown to contribute to AbdA-mediated *Dll* repression [13], [15], [29]–[31]. In addition, the region separating the HX from the HD, termed the linker region (LR), was shown to control the efficiency of *Dll* repression by Ubx and AbdA [14]. Sequence conservation also includes a QRQA sequences C-terminally adjacent to the HD. Interestingly, this highly conserved sequence follows positions with lower sequence conservation. The sequence is in a position similar to the UbdA motifs, a motif shared by Ubx and AbdA, which is either essential or contribute to *Dll* repression in Ubx and AbdA respectively [13], [15], [31].

To address the functional importance of these regions for AbdB-mediated *Dll* repression, mutations in these domains were engineered in the *Drosophila* protein and transgenic lines allowing the expression of these variants under UAS control were generated. prd-Gal4 driven expression showed that single mutation of the W residue, or combined mutation of several residues within this region (TG, EWTG) do not alter AbdB potential to repress DME activity (Figure 6 and Figure S6). We also mutated the position that immediately precedes the initiation of the HD, that is flanked by conserved residues, and whose identity, an S in *Drosophil*a or a T in some other arthropods, may suggest a potential for post-translational modification by phosphorylation. As other HD N-terminal located mutations, this mutation does not affect AbdB repressive potential (Figure 6 and Figure S6). In contrast, altering the QR sequence lying C-terminal to the HD (AbdB*^Cter^*) results in a strong reduction of the AbdB repressive potential (Figure 6 and Figure S10).

We next investigated the importance of AbdB HD sequences for DME repression. We first aimed at generating mutations that would alleviate AbdB DNA binding. Based on DNA contacts seen in the HoxA9-Pbx1-DNA crystal structure [16], we targeted position 50 and 51 the HD recognition helix 3 (Figure 6 and Figure S10). Individual mutation of these positions (AbdB*^H3a^*, AbdB*^H3b^*) resulted in a complete loss of repressive activity, demonstrating the essential character of AbdB DNA binding for proper *Dll* repression (Figure 6 and Figure S10).

We then generated mutations in the HD N-terminal arm that in some Hox proteins was shown to be crucial for functional specificity [32]–[34]. This region of the HD contains all paralogue specific signatures of posterior and central class Hox proteins, defined by positions whose identity is shared by all members of a paralogue group, but not by any other paralogue group [3]. A first set of mutations aimed at altering the posterior class specific signature was achieved by changing two lysines in positions 3 and 4 of HD to alanines (AbdB*^KK^*). Results showed that AbdB*^KK^* fails to properly repress DME, with a loss of 60% of its repressive potential (Figure 6 and Figure S10). We next asked if endowing the AbdB N-terminal arm with the specificity of central Hox protein (Ubx and AbdA) would allow a significant restoration of the repressive function. Central Hox proteins display a paralogue specific signature made of three residues, G,Q, and T, at positions 4, 6 and 7 respectively. These positions were changed to the identity of central Hox proteins, which in part compromise the posterior class signature, while grafting the central class signature (AbdB*^CEN^*). Results showed that AbdB*^CEN^* has a very weak repressive potential, even lower than that of AbdB*^KK^* (Figure 6 and Figure S10), indicating that HD paralogue specific signatures are not sufficient to confer DME repression. This result is consistent with the contribution of sequences outside the HD for Ubx/AbdA-mediated *Dll* repression [13], [15], [31].

Taken together, this functional dissection of AbdB protein domain requirement for DME repression demonstrates the dispensability of sequences immediately N-terminal to the HD (the HX and linker region), establishes a contribution of the HD N-terminal arm and residues immediately C-terminal to the HD, and reveals a strict requirement for AbdB DNA binding.

### Endowing the central Hox protein Ubx with a posterior AbdB like repressive mode

**Figure 7:**
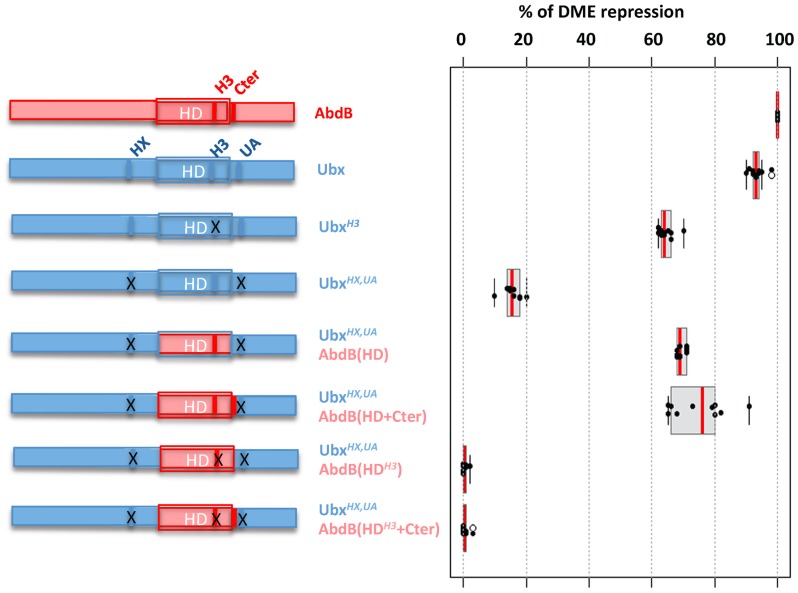
Requirement of protein domains in AbdB/Ubx chimeric proteins for DME repression. Left part of the figure depicts wild type and mutated variants of Ubx (blue), including mutations (indicated by crosses) in the HX, and UbdA (UA) domains as previously described [15] and in helix 3 (Q50 to K50) [55]. AbdB protein sequences, including or not the QR domain, either with a wild type or mutated helix 3, is represented in red. Effects of the mutations on the repressive activity of AbdB on DME are displayed in a box plot representation. Switching the Ubx HD by that of AbdB endows the chimera with a posterior AbdB like dependent mode of DME repression. Illustration of data is given in Figure S11.

Mutations of helix 3 of the HD at positions 50 and 51, known to provide strong DNA contacts in the DNA major groove, highlight the strict requirement of AbdB DNA binding for proper *Dll* repression. To address if the conclusion also holds true for central Hox proteins, we investigated the requirement of position 50 within helix 3 of the Ubx HD for proper DME repression. Mutation of position 50 of the HD was previously shown to be essential for Ubx binding to DMX-R [35], [36]. Yet, expression of this helix 3 mutated Ubx protein (Ubx*^H3^*) showed that it still represses DME, with a limited loss (30%) of repressive potential. (Figure 7 and Figure S11). Taken together with the strict requirement in AbdB of residues contacting DNA within AbdB helix 3, we concluded that a major difference in the mode of DME repression by AbdB and the central Hox protein Ubx lies in the requirement/dispensability of DNA binding in the absence of the Exd and Hth TALE cofactors.

We next studied whether we could endow the Ubx protein with an AbdB like mode of DME repression. We used as a recipient protein a Ubx protein bearing a UbdA mutation, as well as a mutation in the HX, which slightly enhance the loss of repressive potential resulting from the UbdA mutation [15]. The first chimera consisted in swapping the Ubx HD by that of AbdB (Ubx*^HX,UA^*AbdB(HD)), which significantly restored repressive potential (68% instead of 17% for Ubx*^HX,UA^*; (Figure 7 and Figure S11)). As we found that sequences immediately Cter adjacent to the AbdB HD contributed to full AbdB repressive activity, we also generated a chimera which in addition included the AbdB QRQA Cter residues. This addition did however not significantly enhanced the repressive activity of the chimera (71% for Ubx*^HX,UA^*AbdB(HD+Cter) instead of 68% for Ubx *^HX,UA^*AbdB(HD); (Figure 7 and Figure S11). These results suggest that swapping the HD is sufficient to endow Ubx with an AbdB like repressive mode. To confirm this, we next asked if the Ubx*^HX,UA^*AbdB(HD) and Ubx*^HX,UA^*AbdB(HD+Cter) use an AbdB like mode of repression, by investigating if DNA binding is critical for the activity of these chimeras. Mutations of position 51 of the HD within the context of these two chimeras were generated, and the resulting chimeras were assayed for DME repression. Results showed that Ubx*^HX,UA^*AbdB(HD*^H3^*) and Ubx*^HX,UA^*AbdB(HD*^H3^*+Cter) are fully deficient in DME repression (Figure 7 and Figure S11), indicating that these chimeras use a mode of repression that strictly requires DNA binding. We concluded that swapping the HD is sufficient to endow Ubx with an AbdB mode of DME repression.

## Discussion

**Figure 8:**
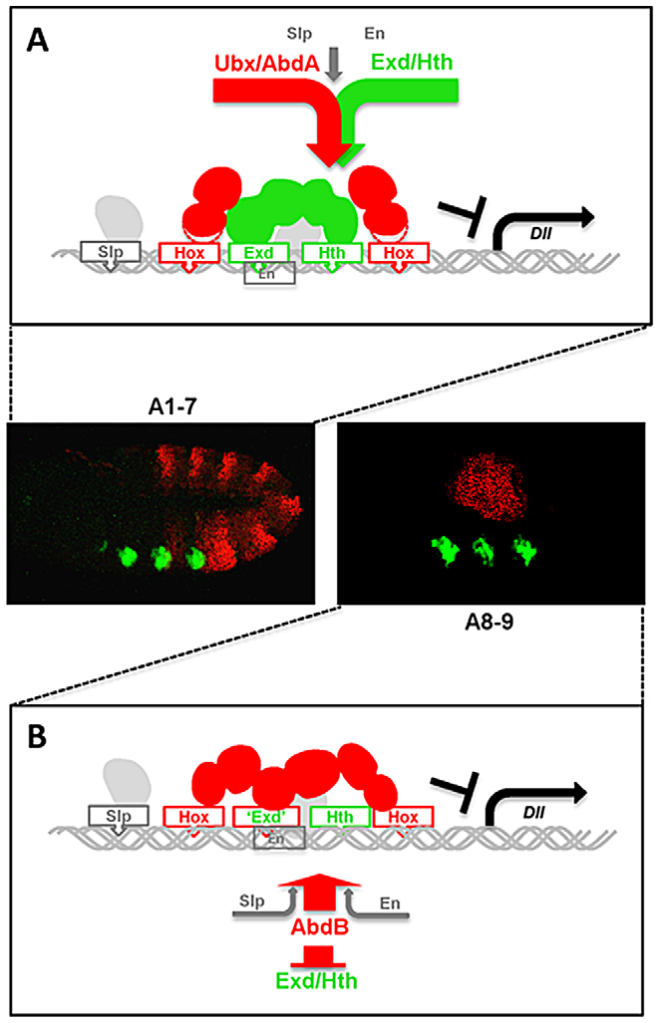
Models for distinct Hox cofactor partnership for *Dll* repression. (A) Model for repression of *Dll* by Ubx/AbdA in anterior abdominal segments A1-7. Repression relies on the assembly of a Hox/Exd/Hth protein complex [20]. DNA binding by Hox proteins is not essential (depicted by a dashed delineated pink zone of contact between the Hox protein and the DNA), as supported by the limited loss of repressive activity of a DNA binding deficient Ubx protein (Figure 7), and by the limited dereprepression associated to mutation in Hox binding sites. The non-essential character of Hox DNA binding may result from acting in a context of a multiprotein complex containing two additional DNA binding proteins (Exd and Hth). (B) Model for repression of *Dll* by AbdB in posterior abdominal segments in A8-9. AbdB represses Exd and Hth, and consequently act without the aid of Exd and Hth to repress *Dll*. This difference likely imposes a strict requirement for AbdB DNA binding for efficient repression.

Using the repression of the limb promoting gene *Dll*, we have investigated how highly divergent central (Ubx/AbdA) and posterior (AbdB) Hox proteins perform an identical function. The comparison of *Dll* regulatory cis requirements, use of cofactors, and requirements in Hox protein intrinsic domains demonstrate distinct modes of *Dll* regulation, highlighting usage of distinct molecular strategies by divergent Hox proteins to achieve common biological functions (Figure 8).

### Distinct intrinsic determinants within Ubx and AbdB for *Dll* repression

As AbdB lacks motifs known in Ubx and AbdA to mediate *Dll* repression [13]–[15], [31], we searched for AbdB intrinsic determinants responsible for *Dll* repression in A8 and A9. Our results highlight that, as for Ubx, a short sequence immediately Cter to the HD is required for full repression. The Cter peptides in Ubx/AbdA and AbdB are however distinct, and serve different functions: in the case of Ubx, its role is to recruit Exd, while in AbdB its role must be different as *Dll* repression by AbdB does not require Exd activity.

Most strikingly, we found that mutations that alleviate DNA binding results in different outputs in Ubx and AbdB. A Ubx protein that lacks DNA binding activity still represses *Dll* efficiently, while a DNA binding deficient AbdB protein does not. We interpret this difference as resulting from Ubx binding DNA within the context of a multiprotein complex involving the Exd and Hth proteins [14], [15], [19], [20], which are also DNA binding proteins that may compensate the loss of Ubx binding to DNA. In contrasts, AbdB binds DNA in the absence of these potential compensating partners. Such compensatory roles were recently reported for other Hox/TALE complexes [37]. The importance of the AbdB HD for *Dll* repression was further demonstrated by the ability of a HD swap between Ubx and AbdB. Thus, the intrinsic requirements for Ubx and AbdB mediated *Dll* repression are different, supporting that distinct molecular mechanisms are used for *Dll* repression.

### Different use of Exd and Hth cofactor for *Dll* repression by Ubx and AbdB

We tested the role of the four protein partners previously identified as crucial for *Dll* repression by Ubx and AbdA [20]. En and Slp expressed at similar levels in the anterior and posterior abdomen are required for AbdB-mediated repression of *Dll*, while Exd and Hth are absent or present at very low levels in the posterior abdomen where AbdB represses *Dll*. The differential expression of Hth and Exd in the abdomen results from a strong down regulation by AbdB, while Ubx and AbdA cause weaker effects on Hth and Exd expression (this study and [23]). These distinct properties of central and posterior class Hox proteins Ubx/AbdA and AbdB allow to set up a pattern where Hth/Exd are present in the anterior abdomen, in places where *Dll* repression [19] as well as other Ubx/AbdA functions [30], [38]–[40] require these cofactors, and absent or at weak/barely detectable levels in the posterior abdomen. Taken together with the dispensability of Hth and Exd for proper posterior spiracle morphogenesis [39], [41], this indicates that AbdB, at least in the embryo, functions without the aid of the Hth and Exd cofactors.

The dispensability of Exd/Hth needs to be correlated with the effects of mutations in the Exd and Hth binding sites which in DMX (or DME) results in de-repression all abdominal segments including in A8 and A9 [19], [20]. Mutation of the Exd binding sites strongly reduces AbdB binding to DMX-R, providing a basis for derepression in the posterior abdomen. Mutation of the Hth binding site does not impact on AbdB binding, suggesting it may serve binding to a protein that remains to be identified. Of note mutations of the “Hth binding sites” result in posterior specific de-repression [20], suggesting that it may affect binding/function of the En compartment specific repressor.

Beyond dispensability, the absence of Exd and Hth in the embryo may be required for proper AbdB function. This view is supported by our *in vitro* EMSA's on DMX-R showing a competition effect of Hth and Hht/En/Exd complexes on AbdB binding, and by de-repression of *Dll* in posterior segments A8 and A9 following increased levels of Hth expression. Functional antagonism between AbdB and the TALE cofactors Exd and Hth is further demonstrated in the specification of several AbdB-dependent specific features, including the posterior spiracle and the suppression of ventral denticle belts [24]. While this set of data support antagonistic AbdB/Exd/Hth partnership, cooperative partnership may also exists, as suggested by the co-expression of AbdB and Exd/Hth in the genital disc [42] and the assembling of AbdB-Exd-Hth-DNA complexes in vitro [43]. The functional significance of AbdB/TALE cooperative partnership remains however to be established, and its contribution to AbdB mode of action clarified, as this partnership decreases the binding selectivity of AbdB, while it increases that of anterior and central Hox proteins [43].

Our results also provide additional support for developmental functions performed independently by Hox proteins and their usual cofactors Exd and Hth. In *Drosophila*, some aspect of Hox protein function do not require Exd, including the function of the central Hox protein Ubx in specifying haltere development [44] and reversely, Exd and Hth have functions that are not Hox dependent, as illustrated by the control of embryonic trachea development [45] and antennal identity [46]. Such independent functions have also been described in vertebrates, for example during face morphogenesis, a situation where Pbx proteins acts in a Hox-free domain [47]. Altogether, this emphasizes that Hox proteins and their cofactors may use in a context specific manner multiple mode of interactions, ranging from cooperativity to dispensability.

### Modes of central and posterior class Hox DNA binding and plasticity of *Dll* cis regulatory sequences

Although generally conserved, the mode of HD/DNA contacts significantly varies between anterior/central and posterior paralogue groups [48]. In particular, it was shown that posterior paralogue proteins possess enhanced DNA binding affinities that in part result from the ability to make extensive contacts with the DNA backbone. Hox proteins of the anterior/central paralogue bear residues critical for functional specificity within the N-terminal arm of the HD [32]–[34]. Proper folding of the N-terminal arm necessary for efficient binding requires the interaction with Exd [28]. Our results are consistent with such distinct mode of DNA binding: AbdB efficiently binds the *Dll* enhancer on its own, while binding by Ubx or AbdA requires the assistance of the Exd and Hth cofactors.

Taken together with previous data on *Dll* regulation by Ubx and AbdA [13]–[15], [19]–[21], [31], our results indicate that *Dll* repression in abdominal segments needs to accommodate the repression by different molecular complexes. This relies on the plastic usage of the same *Dll* cis sequences in the anterior and posterior abdomen. First, Hox binding sites may accommodate binding by Hox proteins displaying significantly divergent mode of DNA binding, as shown by the use of Hox1 and Hox2 binding site for Ubx/AbdA and AbdB DNA binding. Second, the same cis sequence binds distinct proteins, as shown for the initially labeled “Exd binding site” that also mediates AbdB binding to DMX-R. The current mode of *Dll* cis sequence usage may reflect the evolutionary history of *Dll* repression: *Dll* repression may have initially been achieved by AbdB, and have later been extended to repression by Ubx/AbdA by the acquisition/cooptation of Exd and Hth binding sites, enabling Ubx and AbdA to bind the enhancer, despite having a HD not optimized for efficient binding to the *Dll* gene.

## Materials and Methods

### Fly stocks, transgenesis, gain-of-function experiments, and in situ transcripts hybridization

The following stocks were used for the study: UAS-Ubx::HA [49], UAS-Ubx, UAS-AbdA, UAS-AbdBm [50], UAS-AbdBr (received from James Castelli-Gair Hombria), UAS-Slp and UAS-En (received from Richard Mann), UAS-Exd and UAS-Hth (received from Natalia Azpiazu), DMX-lacZ [20], prd-Gal4 [51] and arm-Gal4 [52].

Transgenic flies were generated using P-element germline transformation either in yw flies [53] or in flies with site specific integration sites (attb) [54]. All constructs were cloned in pUAST vector and sequence verified.

AbdB variants and AbdB/Ubx chimeras were generated using the SOE method, starting form UbxIa and AbdBm cDNAs, and cloned into pUAST vector (EcoRI, XhoI). Primers were as follows:

AbdB variants:

AbdB m (5′ AAAAGAATTCATGCAGCAGCACCATCTGCA; 5′ CGGCGGTTCTACGTGGTTGAGCTCAAAA)

AbdB ^w^ (5′ CCCGGACTGCACGAGGCAACGGGC; 5′ GGGCCTGAGGTGCTCCGTTGCCCG)

AbdB ^TG^ (5′ GAGTGGGCAGCACAGGTGTCCGTC CG; 5′ CCTGACGTGCTCACCCGTGTCCAC)

AbdB ^EWTG^ (5′ AATCCCGGACTGCACGCAGCAGCCGCACAGGTG; 5′ TTAGGGCCTGACGTGCGTCGTTGG

CGTGTCCAC)

AbdB ^S^ (5′ GGTCAGGTGGCAGTCCGGAAAAAGCGC; 5′ CCACTGCACCGTCAGGCCTTTTTCGCG)

ABdB ^KK^(5′ CAGGTGTCCGTCCGGGCAGCACGCAAGCC5; 5′ GTCCACAGGCAGGCCCGTCGTGCGTTCGG)

AbdB ^CEN^ (5′ GTCCGGAAAGGACGCGAAACCTACTCCAAG; 5′ CAGGCCTTTCCTGCGCTTTGGATGAGGTTC)

AbdB ^H3a^ (5′ ATATGGTTCGCAAATCGCCGCATG; 5′ CAGTTCTATACCAAGCGTTTAGCC)

AbdB ^H3b^ (5′ ATATGGTTCCAGGCACGGCGGATGAAGAAC; TATACCAAGGTCCGTGCCGCCTACTTCTTG)

AbdB C^ter^(5′ TCACAGGCAGCACAGGCGAATCAG; 5′ TTCTTGAGTGTCCGTCGTGTCCGC)

Ubx/AbdB chimeras (Ubx ^HXUA^ and AbdBm were used as templates):

AbdB HD amplification (5′ ACAAATGGTCTGGTCCGGAAAAAG; 5′ GATCGCCTGTGAGTTCTTCTT)

Ubx N-Ter amplification (5′ AAAAGAATTCATGAACTCGTACTTT; 5′ TGTTTACCAGACCAGGCCTTTTTC)

Ubx C-Ter amplification (5′AAGAAGAACTCACAGGCGATCAAGGTG; 5′ GTGAATCTAGTCGAGCTCAAAA)

For Ubx ^HXUA^(AbdB ^H3^) template used for AbdB HD amplification was AbdB ^H3b^.

For Ubx ^HXUA^ (AbdB HD+Cter) and Ubx ^HXUA^ (AbdB HD^H3^+Cter), the procedure was similar using the AbdB HDCter 3′ (5′ TTCTTCTTGAGTGTCGCGGTCCGGCTCTTCGTC) instead of (5′ GATCGCCTGTGAGTTCTTCTT).

P insertions were genetically mapped. For each variant, two lines were crossed with the prd-Gal4 and arm-Gal4 driver at 22, 25, or 29°C. Collected embryos were stained with anti-Ubx (FP3.38, dilution 1/1000), anti-AbdB(DSHB, I/10) or anti-HA tag (Eurogentec, dilution 1/1000) to select the conditions (line and temperature) that result in expression levels similar (+/−15%) to Ubx and AbdB wild-type levels in A1 and A8, respectively [14], [31]. Levels of Ubx and AbdB in wild-type embryos were assessed in a sized region in the middle of A1 and T2, respectively. The mean luminosity values for these regions were established by using the AxioVision LE4.5 measurement tool. *Hth* and *Dll* Digoxigenin RNA-labelled probes were generated by in vitro transcription from plasmid containing *hth* and *Dll* cDNA. RNA in situ hybridization were performed according to standard methods.

### Quantification of DME repression in gain of function experiments and of DMX bearing cis mutations derepression

Embryo collections and immunostaining of embryos were performed according to standard procedures. Quantification of DME repression was achieved following anti-β-galactosidase immunostainings (rabbit anti-β-galactosidase (Cappel, 1/1000) by using the same DME-lacZ insertion. The levels of DME enhancer repression were estimated by quantifying the surface reduction in T2 of the DME-positive cell cluster by using the AxioVision LE4.5 measurement tool. Quantification was done on five individual experiments for each genotype. In case of de-repression observed in DMX binding sites mutants, the area of de-repressed β-gal was measured in A1 and A8 in at least 10 embryo's. The average was taken and compared to levels of DMX-lacZ de-repression levels observed in A1 and A8 segments in *Df P9* (BX-C mutant) embryos.

### Protein, protein expression, and EMSA

AbdB variants and AbdB/Ubx chimeras generated as described above were cloned into pcDNA3 (EcoRI, XhoI) for protein synthesis. Exd and Hth were full-length, and En protein was lacking the 60 N-terminal amino acids. AbdB and AbdB with HD mutations were cloned in pCDNA3 vector and sequence verified. Proteins were produced with the TNT (T7)-coupled *in vitro* transcription/translation system (Promega). The following double stranded oligos (only one strand is specified) spanning the *Dll* repressive sequences were used:

DMX-R containing Slp, Hox1, Exd, En, Hth and Hox2 binding sites:

GACAATATTTGGGAAATTAAATCATTCCCGCGGACAGTTTTATAGTGC

DIIRL: containing Hox1, Exd, En,and Hth and Hox2 binding sites: TTTGGGAAATTAAATCATTCCCGCGGACAGTTTTATAGTGC

DIIR containing Hox1, Exd, En,and Hth binding sites: TTTGGGAAATTAAATCATTCCCGCGGACAGT

Mutations in Hox1, Hox2, Exd and Hth were previously described (Gebelein et al, 2004) and are:

Hox1: AAATTAA to AAGCCCG

Hox2: TTTATAG to GGGCTAG

Exd: AAATCAT to AAAGGAT

Hth: GGACAG to GGCCGG

## Supporting Information

Figure S1 Ectopic expression of AbdBm represses thoracic *Dll* expression. A) Embryos stained for the *Dll* transcript (red), showing expression in the thoracic and head segments. B) prd-Gal-4 driven anterior ectopic expression of AbdBm (green) results in *Dll* (red) repression in thoracic segment T2 and head segment (arrowhead).
(TIF)

Figure S2 AbdB, but not Ubx, represses Exd expression. A) Embryos co-stained for Exd (red) and AbdB (green). Arrowheads highlight segments A3 and A8. Upper panels: wild type embryo, decrease of Exd expression is seen from segment A3 and reaches very low levels in A8; Lower panels: embryo lacking AbdB m isoform (AbdB^m3^), displaying posterior Exd accumulation till A8. B) Embryos co-stained for Exd (red) and Ubx (green). Ectopic Ubx expression was driven in every other segments by prd-Gal4. While AbdB ectopic expression (see Figure 5B, left panels) strongly represses Hth expression, Ubx ectopic expression does not.
(TIF)

Figure S3 Binding site requirements for AbdB binding to *Dll* cis sequences. A) EMSA of AbdB on DIIRL (containing binding sites Hox1, Exd, En, Hth and Hox2) and DIIRL mutants for binding sites Hox1, Hox2, Hox 1+2 and Exd. B)Quantification of AbdB binding in EMSA to DIIRL wild type and mutated using the lowest AbdB quantity. Single mutations in Hox1, Hox2 and Exd similarly reduces the efficiency of AbdB binding to DMX-R, while combined mutation of Hox1 and Hox2 results in stronger decrease in DNA binding. C) EMSA on DMX-R (containing binding sites Slp, Hox1, Exd, En, Hth and Hox2) with AbdB, En, Exd, Hth, Exd+En, Exd+Hth, Hth+En and Exd+Hth+En identifying AbdB-DNA and Exd-Hth-En-DNA complexes.
(TIF)

Figure S4 Requirements for Hth/Exd inhibition of AbdB binding to DIIR. EMSA of AbdB on DIIR with various combinations of AbdB, Exd, Hth and truncated HM (HD less) form of Hth, or on DIIR mutated in the Exd (DIIR*^exd^*) or Hth (DIIR*^Hth^*) binding sites. Note that the Exd-mediated release of inhibitory effect seen for full length Hth is lost with the truncated Hth HM protein, and that mutation of the Exd binding site affect the formation of AbdB/DNA complexes.
(TIF)

Figure S5 Quantifications of DMX-R sequence requirements for repression in A1 and A8. A) Schematic representation of DMX-R mutations analysed. DMX lacZ reporter transgenic lines, transcription factor binding site (Slp, Exd, Hth, En and Hox) allocation, and name of DMX-R mutant enhancers are from [19]. DMX-R mutations that result in abdominal repression are highlighted in red. B) Quantification of derepression observed in A1 (blue) and A8 (red) for each DMX-R variant. 100% derepresion was defined by the level of abdominal DMX derepression in embryo deficient for Ubx, AbdA and AbdB m and r isoforms (*Df P9*). Except for the DMX(Hox1S) and DMX(Hox1, 2) binding sites, no difference in derepression was observed between A1 and A8.
(TIF)

Figure S6 Illustrations of DMX-R sequence requirements for repression in A1 and A8. Embryos bearing DMX-R lacZ reporter transgenes mutated in one or multiple binding sites were stained for β-gal. Note that for some of these mutations, a significant variability was observed (see Figure S5) and is not illustrated by a single embryo display as done in this figure. Derepression of DMX-R activity in segments A1 and A8 are magnified.
(TIF)

Figure S7 AbdBm represses the transcription of Hth. A) Embryos stained for the *hth* transcript (red). B) prd-Gal-4 driven ectopic expression of AbdBm (green) results in *hth* (red) repression. Right and left panel shows early (germ band extended) and late (germ band retracted) embryos.
(TIF)

Figure S8 Lack (or low level) of AbdA repressive function on Hth expression. Embryos bearing the DME reporter co-stained for β-gal (green) and Hth (red).
(TIF)

Figure S9 Increasing Hth expression levels in the posterior abdomen allows posterior derepression of DMX in A8 and A9 segments. Embryo bearing the DMX reporter co-stained for β-gal (red) and Hth (green) ubiquitously driven by arm-Gal4. Note that only moderate posterior accumulation of Hth could be reached in posterior abdominal segments. Arrows point to derepression in A8 and A9 segments.
(TIF)

Figure S10 Protein sequence requirements for AbdB-mediated DME repression. Thoracic centered magnifications of embryo bearing the DME reporter co-stained for β-gal (red) and AbdB variants (green) driven by prd-Gal4. Levels of repressive activity of different AbdB murtations on DME were evaluated by defining the ratio of β-gal staining in T2 and T3 (100% of repressive activity was given when the T2/T3 ratio was 1, and 0% when the T2/T3 ratio was O). Quantifications are shown in Figure 6.
(TIF)

Figure S11 Ubx/AbdB chimera protein sequence requirements for DME repression. Thoracic centered magnifications of embryo bearing the DME reporter co-stained for β-gal (red) and Ubx*^H3^* or Ubx/AbdB chimeras (green) driven by prd-Gal4. Levels of Ubx*^H3^* or Ubx/AbdB chimeras repressive activity on DME was evaluated by defining the ratio of β-gal staining in T2 and T3 (100% of repressive activity was given when the T2/T3 ratio was 1, and 0% when the T2/T3 ratio was O). Illustrations for wild type AbdB is given in Figure 1J, 1K, and for Ubx*^HX,UA^* in [15]. Quantifications are shown in Figure 7.
(TIF)
