# Supplementary figures and images for: Distinct Molecular Strategies for Hox-Mediated Limb Suppression in Drosophila: From Cooperativity to Dispensability/Antagonism in TALE Partnership
Source: PLoS Genet. 2013 Mar 7;9(3):e1003307. doi: 10.1371/journal.pgen.1003307 (PMC3591290; doi:10.1371/journal.pgen.1003307)

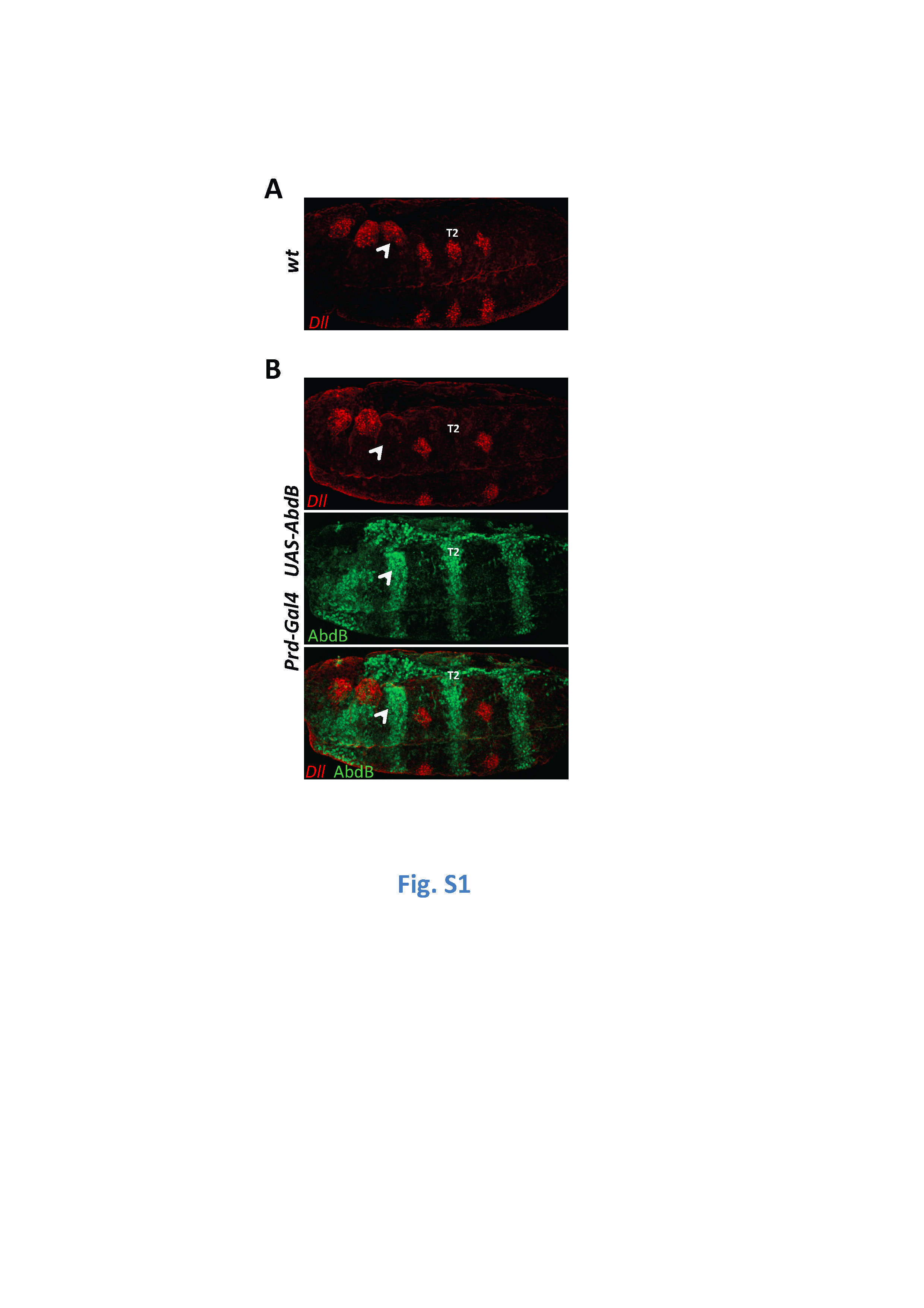

Supplement: Figure S1 — Ectopic expression of AbdBm represses thoracic Dll expression. A) Embryos stained for the Dll transcript (red), showing expression in the thoracic and head segments. B) prd-Gal-4 driven anterior ectopic expression of AbdBm (green) results in Dll (red) repression in thoracic segment T2 and head segment (arrowhead). (TIF) [file pgen.1003307.s001.tif]

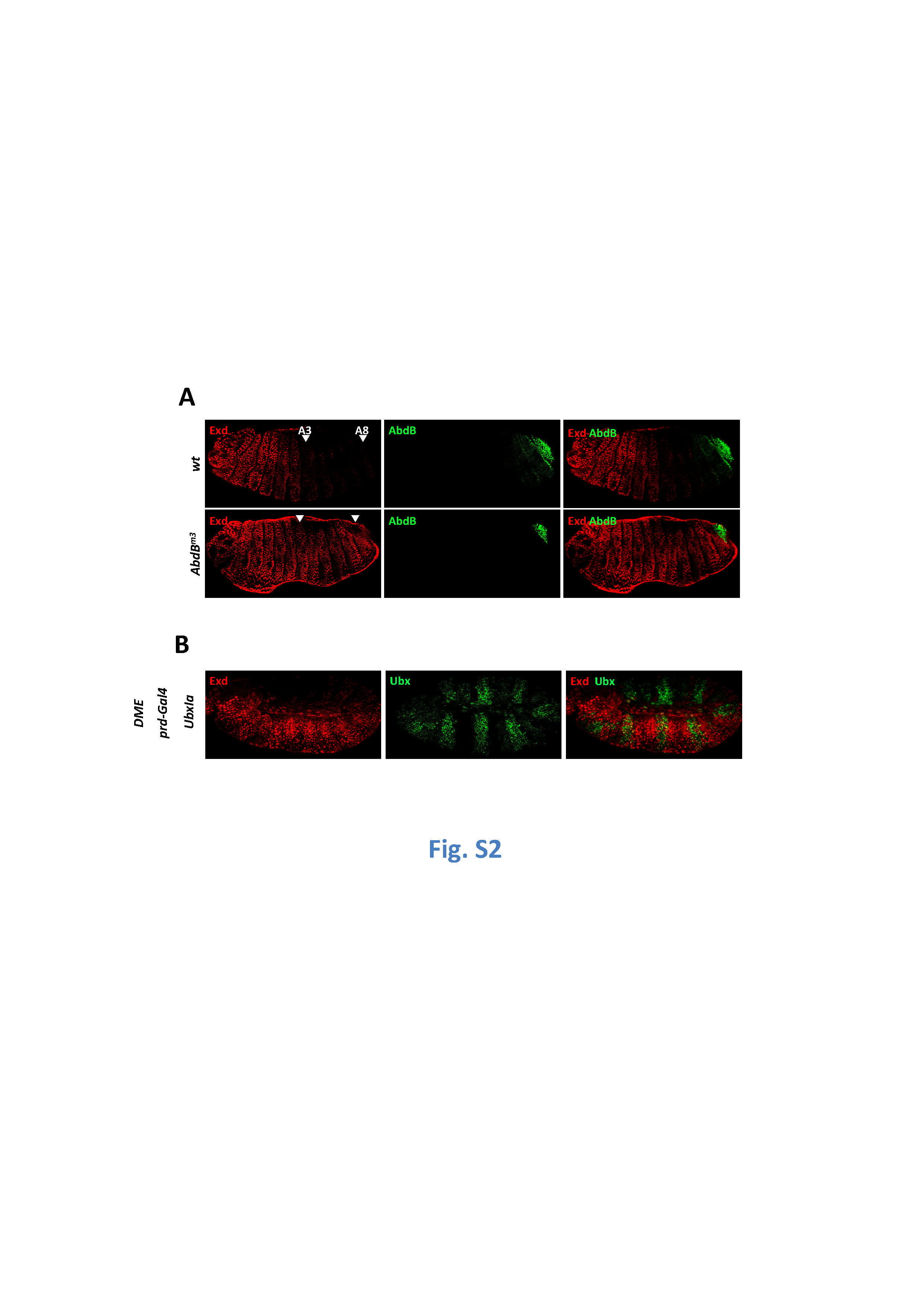

Supplement: Figure S2 — AbdB, but not Ubx, represses Exd expression. A) Embryos co-stained for Exd (red) and AbdB (green). Arrowheads highlight segments A3 and A8. Upper panels: wild type embryo, decrease of Exd expression is seen from segment A3 and reaches very low levels in A8; Lower panels: embryo lacking AbdB m isoform (AbdBm3), displaying posterior Exd accumulation till A8. B) Embryos co-stained for Exd (red) and Ubx (green). Ectopic Ubx expression was driven in every other segments by prd-Gal4. While AbdB ectopic expression (see Figure 5B, left panels) strongly represses Hth expression, Ubx ectopic expression does not. (TIF) [file pgen.1003307.s002.tif]

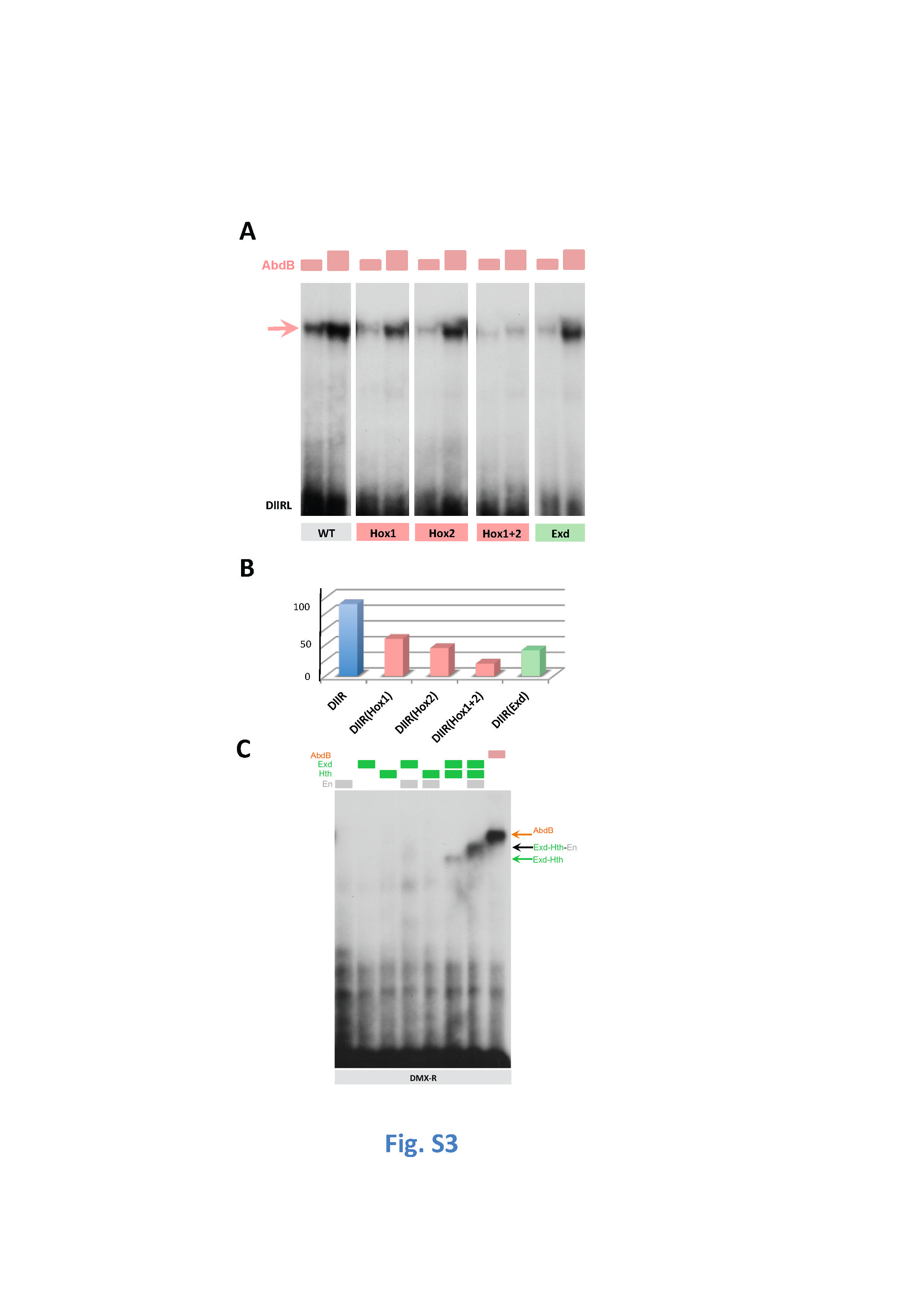

Supplement: Figure S3 — Binding site requirements for AbdB binding to Dll cis sequences. A) EMSA of AbdB on DIIRL (containing binding sites Hox1, Exd, En, Hth and Hox2) and DIIRL mutants for binding sites Hox1, Hox2, Hox 1+2 and Exd. B)Quantification of AbdB binding in EMSA to DIIRL wild type and mutated using the lowest AbdB quantity. Single mutations in Hox1, Hox2 and Exd similarly reduces the efficiency of AbdB binding to DMX-R, while combined mutation of Hox1 and Hox2 results in stronger decrease in DNA binding. C) EMSA on DMX-R (containing binding sites Slp, Hox1, Exd, En, Hth and Hox2) with AbdB, En, Exd, Hth, Exd+En, Exd+Hth, Hth+En and Exd+Hth+En identifying AbdB-DNA and Exd-Hth-En-DNA complexes. (TIF) [file pgen.1003307.s003.tif]

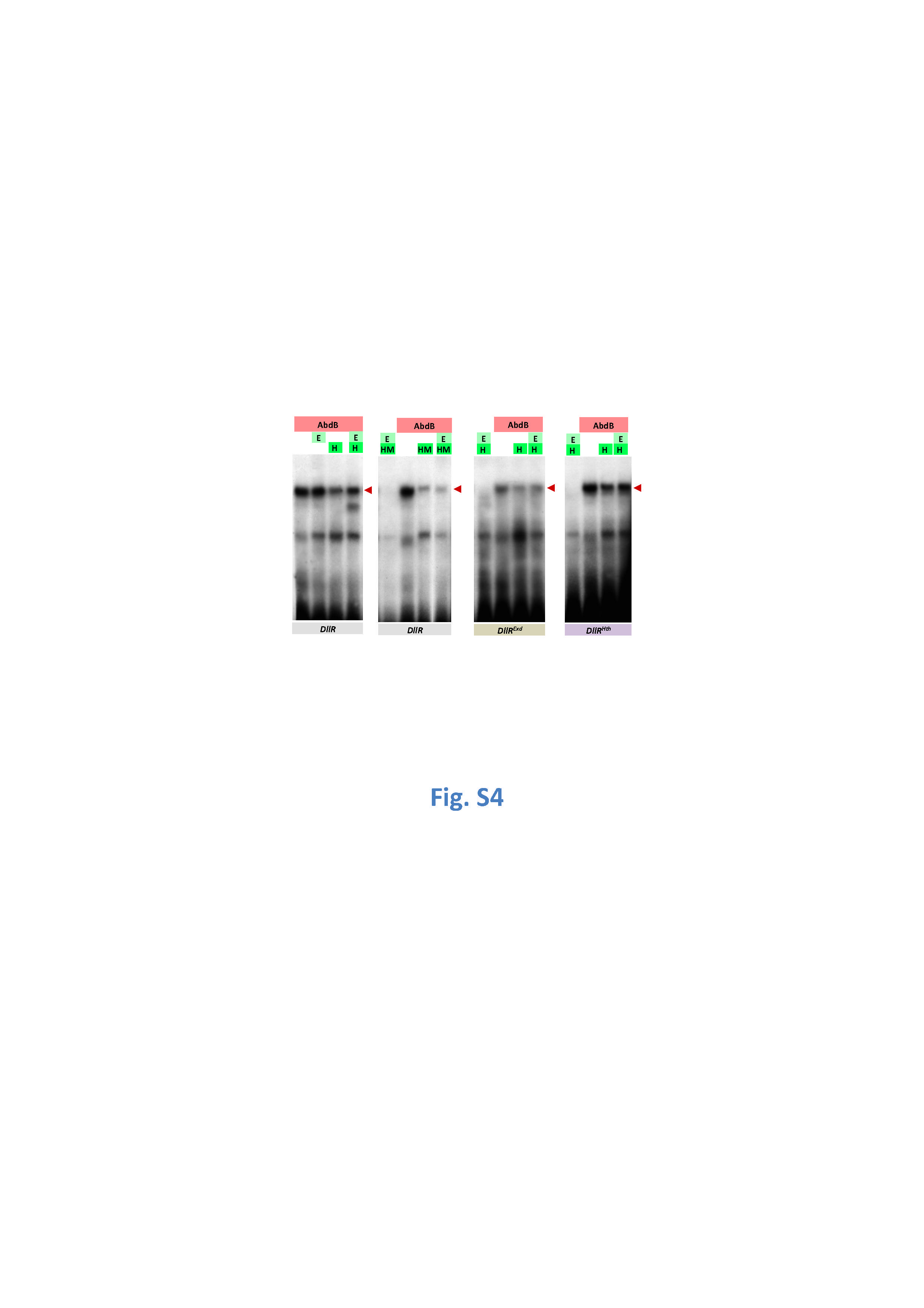

Supplement: Figure S4 — Requirements for Hth/Exd inhibition of AbdB binding to DIIR. EMSA of AbdB on DIIR with various combinations of AbdB, Exd, Hth and truncated HM (HD less) form of Hth, or on DIIR mutated in the Exd (DIIRexd) or Hth (DIIRHth) binding sites. Note that the Exd-mediated release of inhibitory effect seen for full length Hth is lost with the truncated Hth HM protein, and that mutation of the Exd binding site affect the formation of AbdB/DNA complexes. (TIF) [file pgen.1003307.s004.tif]

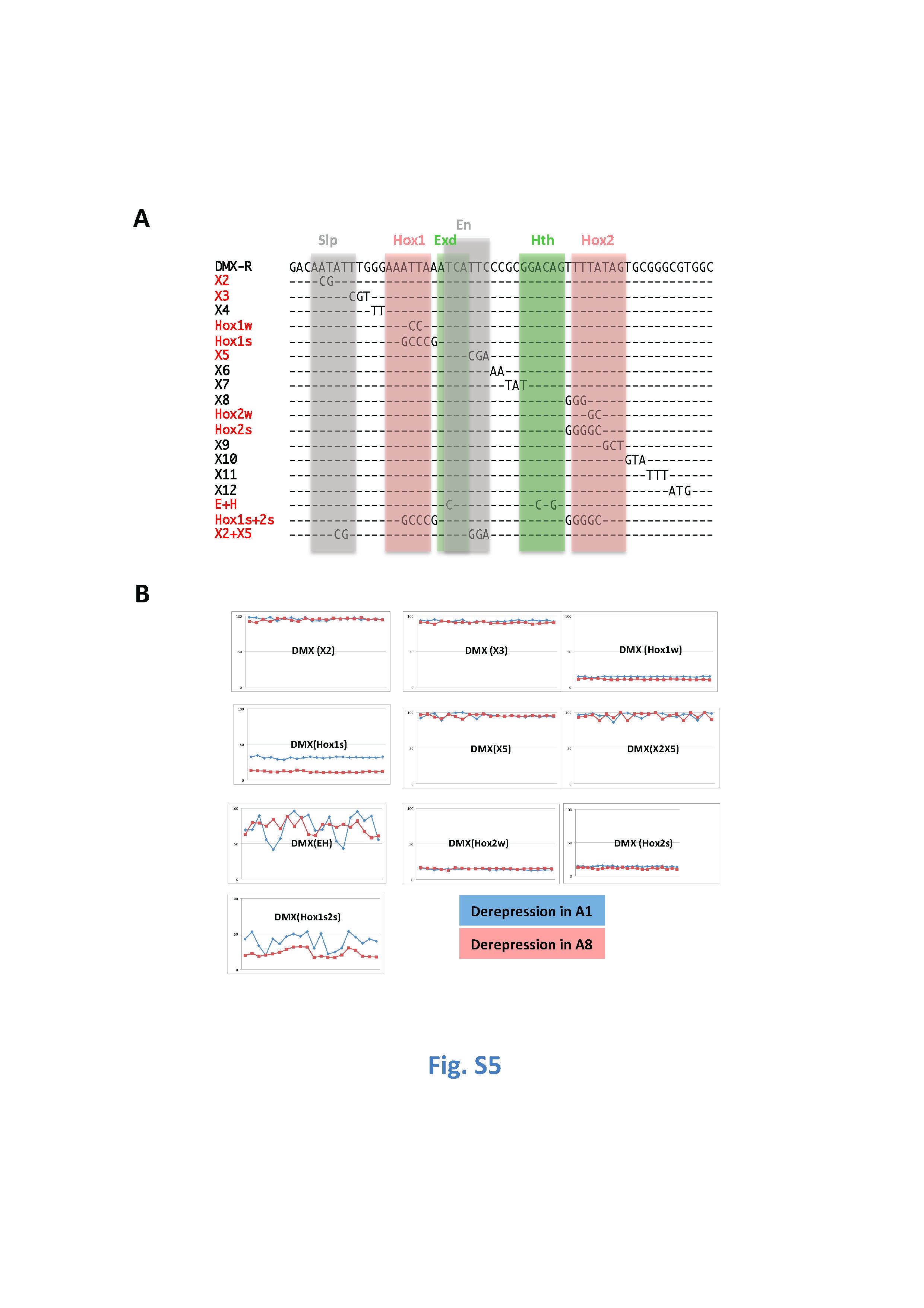

Supplement: Figure S5 — Quantifications of DMX-R sequence requirements for repression in A1 and A8. A) Schematic representation of DMX-R mutations analysed. DMX lacZ reporter transgenic lines, transcription factor binding site (Slp, Exd, Hth, En and Hox) allocation, and name of DMX-R mutant enhancers are from [19]. DMX-R mutations that result in abdominal repression are highlighted in red. B) Quantification of derepression observed in A1 (blue) and A8 (red) for each DMX-R variant. 100% derepresion was defined by the level of abdominal DMX derepression in embryo deficient for Ubx, AbdA and AbdB m and r isoforms (Df P9). Except for the DMX(Hox1S) and DMX(Hox1, 2) binding sites, no difference in derepression was observed between A1 and A8. (TIF) [file pgen.1003307.s005.tif]

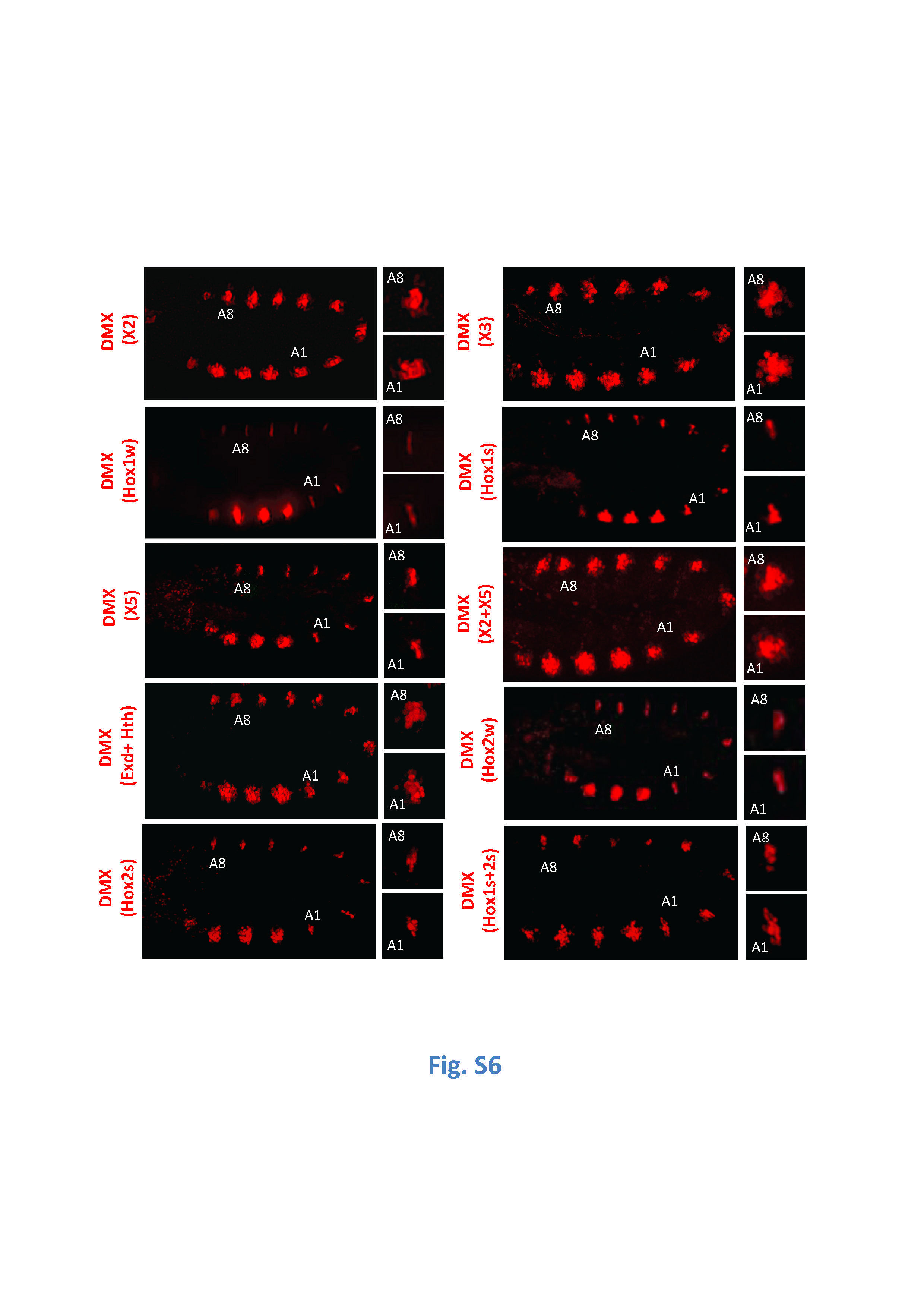

Supplement: Figure S6 — Illustrations of DMX-R sequence requirements for repression in A1 and A8. Embryos bearing DMX-R lacZ reporter transgenes mutated in one or multiple binding sites were stained for β-gal. Note that for some of these mutations, a significant variability was observed (see Figure S5) and is not illustrated by a single embryo display as done in this figure. Derepression of DMX-R activity in segments A1 and A8 are magnified. (TIF) [file pgen.1003307.s006.tif]

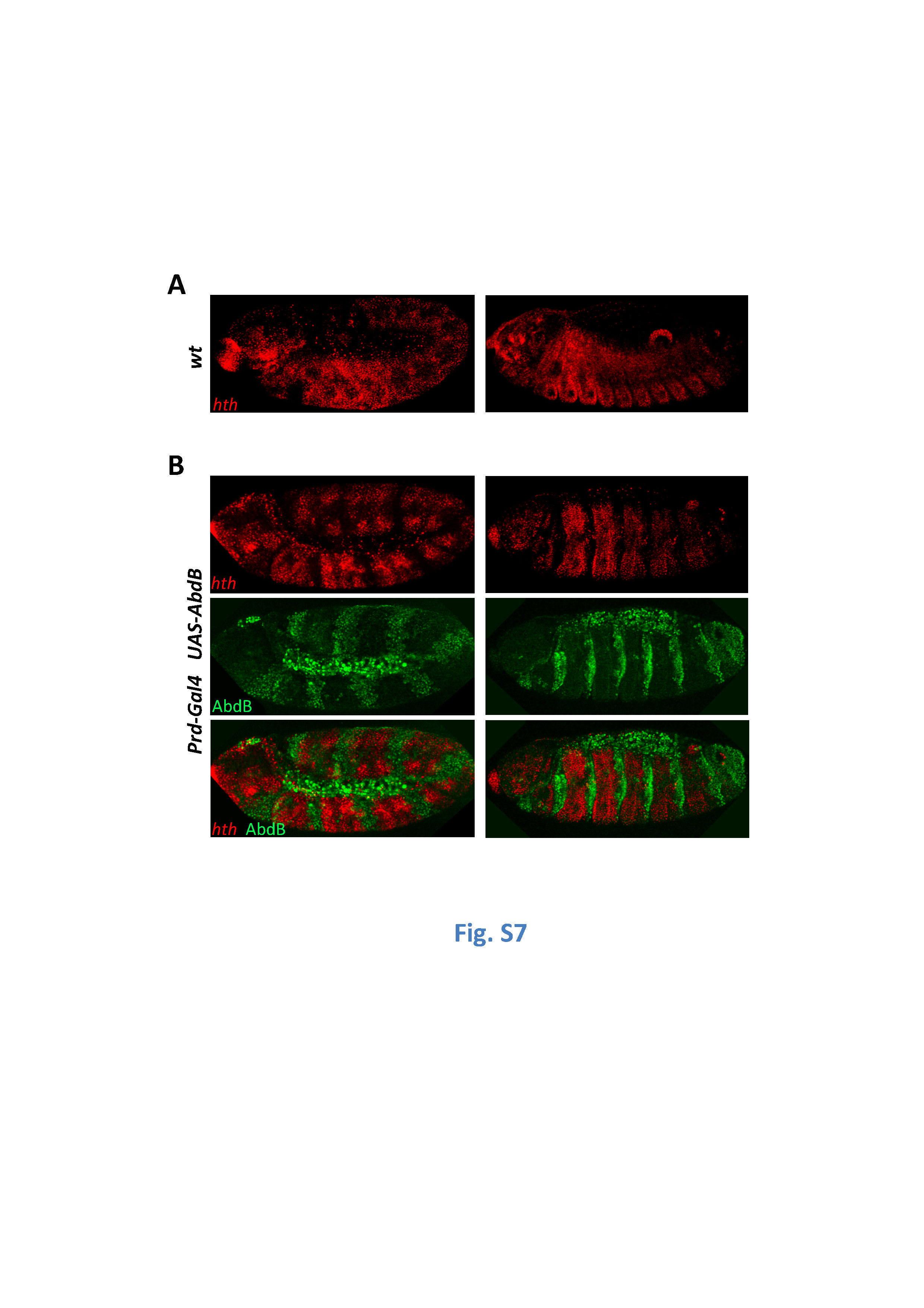

Supplement: Figure S7 — AbdBm represses the transcription of Hth. A) Embryos stained for the hth transcript (red). B) prd-Gal-4 driven ectopic expression of AbdBm (green) results in hth (red) repression. Right and left panel shows early (germ band extended) and late (germ band retracted) embryos. (TIF) [file pgen.1003307.s007.tif]

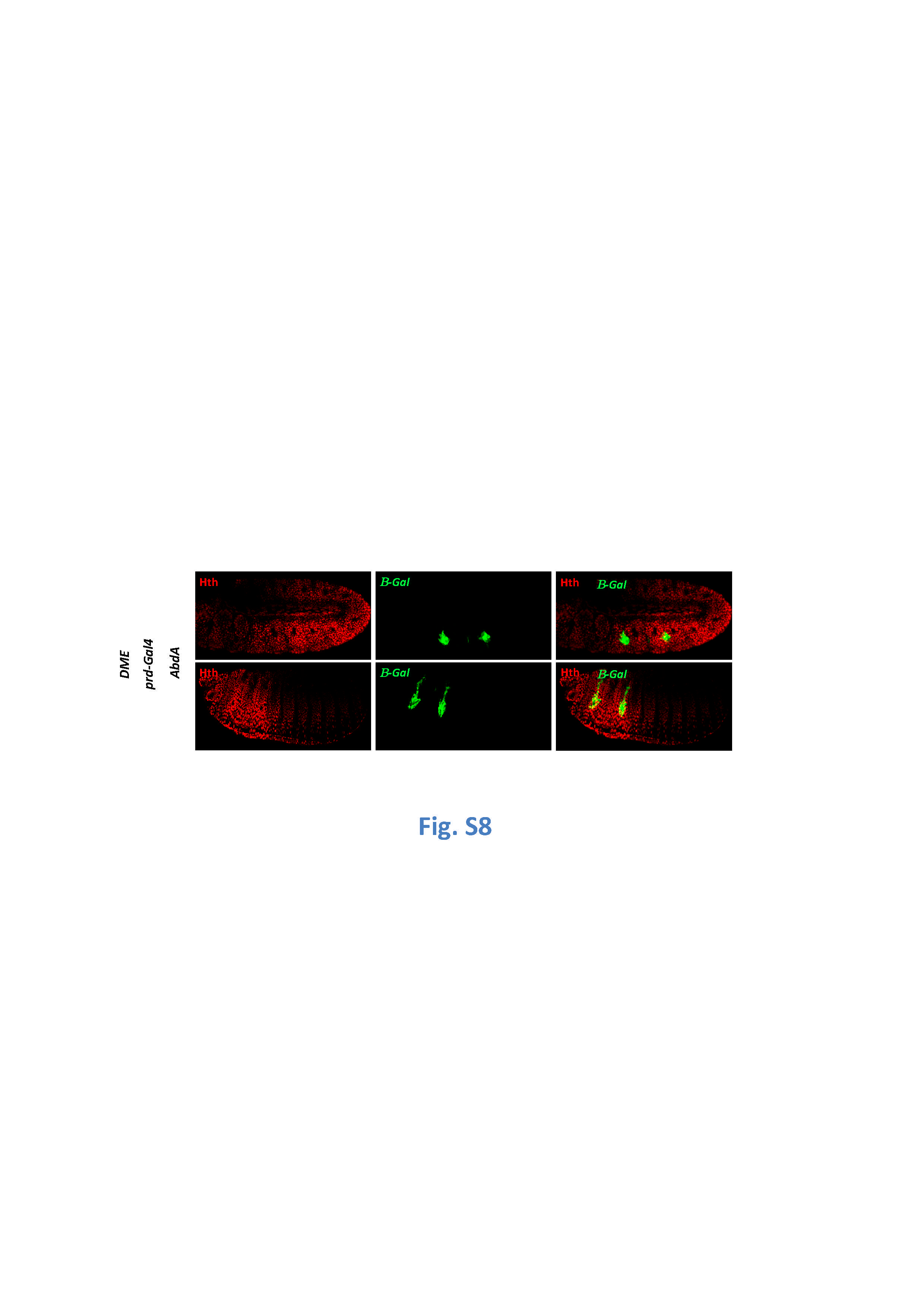

Supplement: Figure S8 — Lack (or low level) of AbdA repressive function on Hth expression. Embryos bearing the DME reporter co-stained for β-gal (green) and Hth (red). (TIF) [file pgen.1003307.s008.tif]

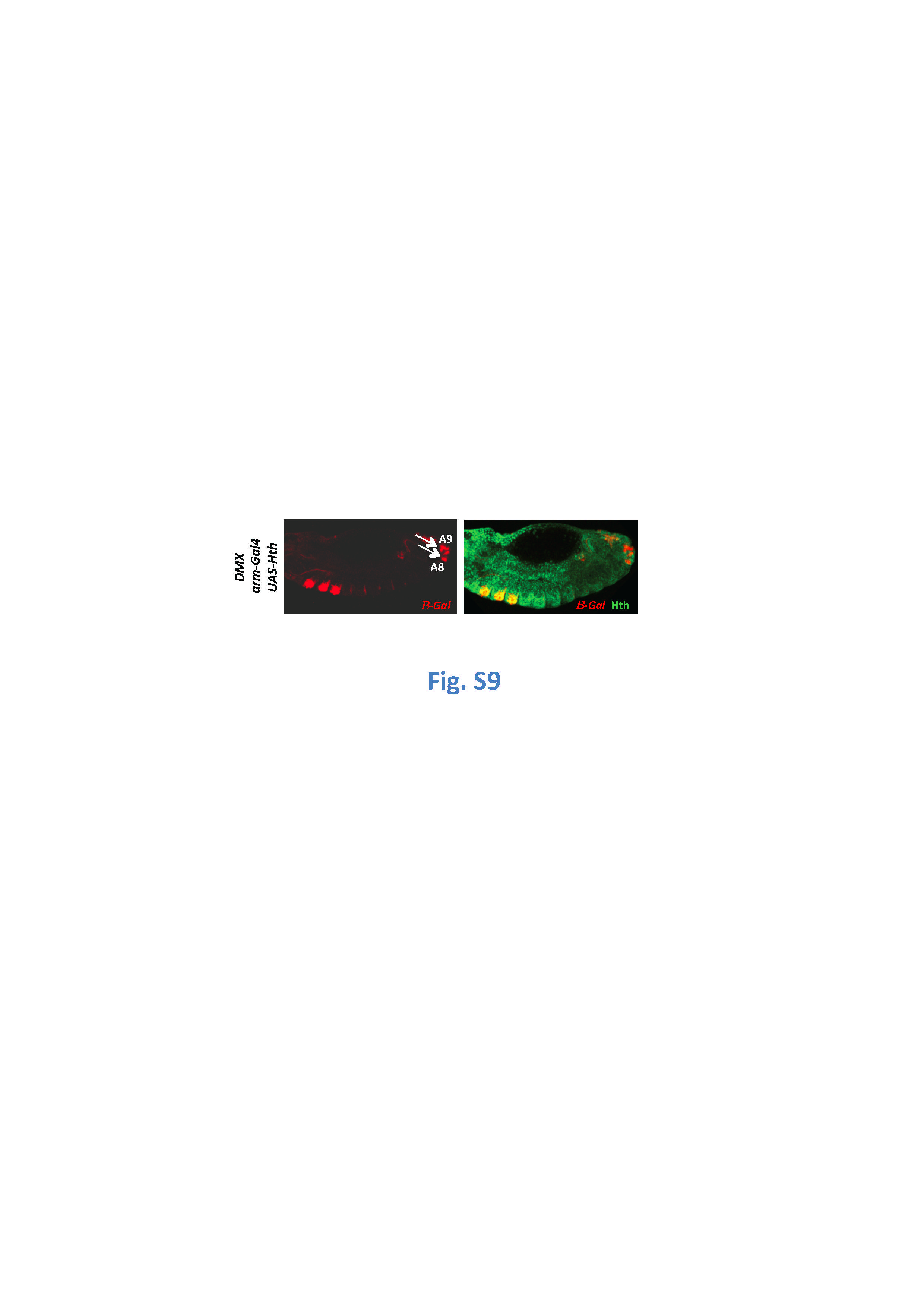

Supplement: Figure S9 — Increasing Hth expression levels in the posterior abdomen allows posterior derepression of DMX in A8 and A9 segments. Embryo bearing the DMX reporter co-stained for β-gal (red) and Hth (green) ubiquitously driven by arm-Gal4. Note that only moderate posterior accumulation of Hth could be reached in posterior abdominal segments. Arrows point to derepression in A8 and A9 segments. (TIF) [file pgen.1003307.s009.tif]

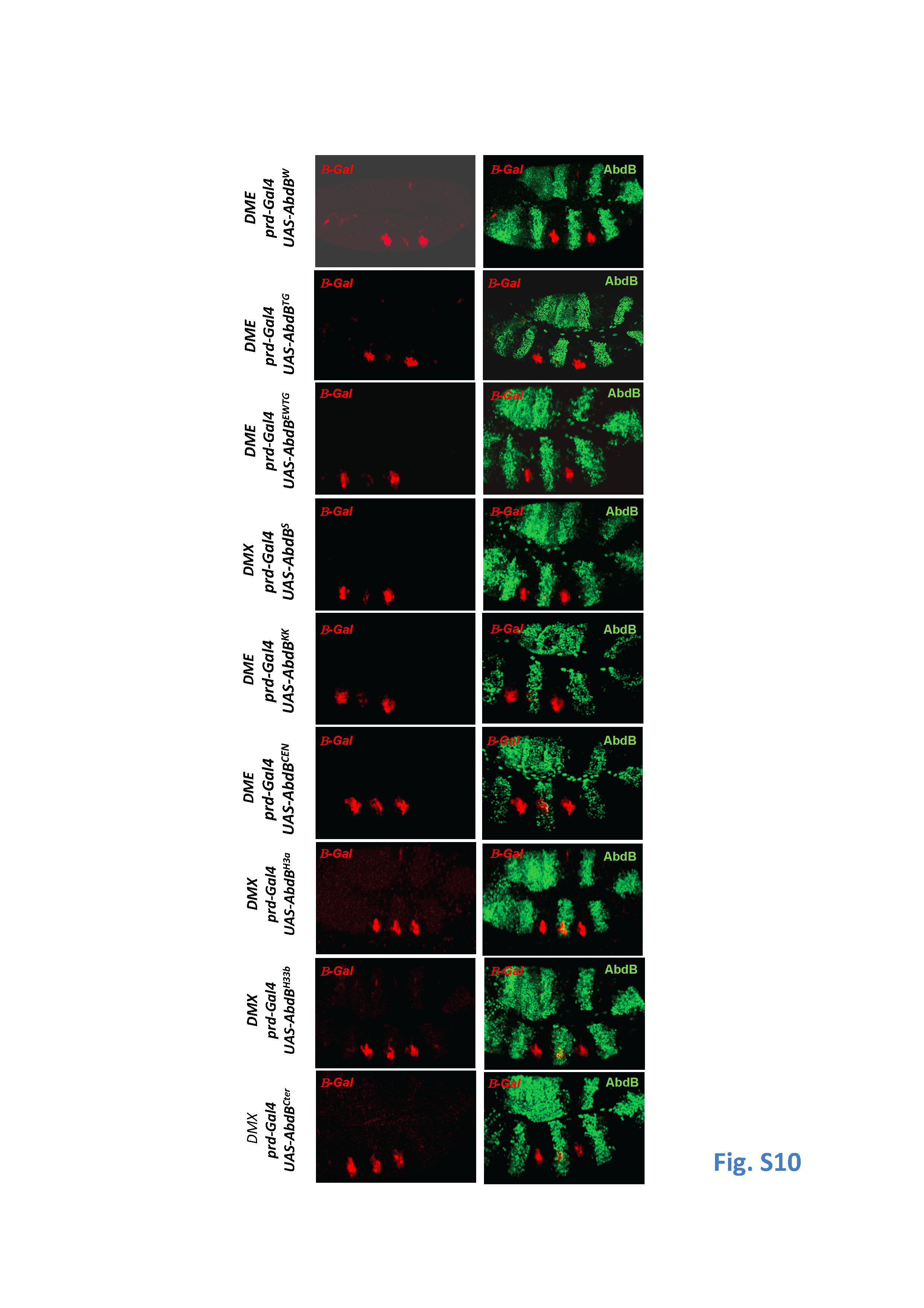

Supplement: Figure S10 — Protein sequence requirements for AbdB-mediated DME repression. Thoracic centered magnifications of embryo bearing the DME reporter co-stained for β-gal (red) and AbdB variants (green) driven by prd-Gal4. Levels of repressive activity of different AbdB murtations on DME were evaluated by defining the ratio of β-gal staining in T2 and T3 (100% of repressive activity was given when the T2/T3 ratio was 1, and 0% when the T2/T3 ratio was O). Quantifications are shown in Figure 6. (TIF) [file pgen.1003307.s010.tif]

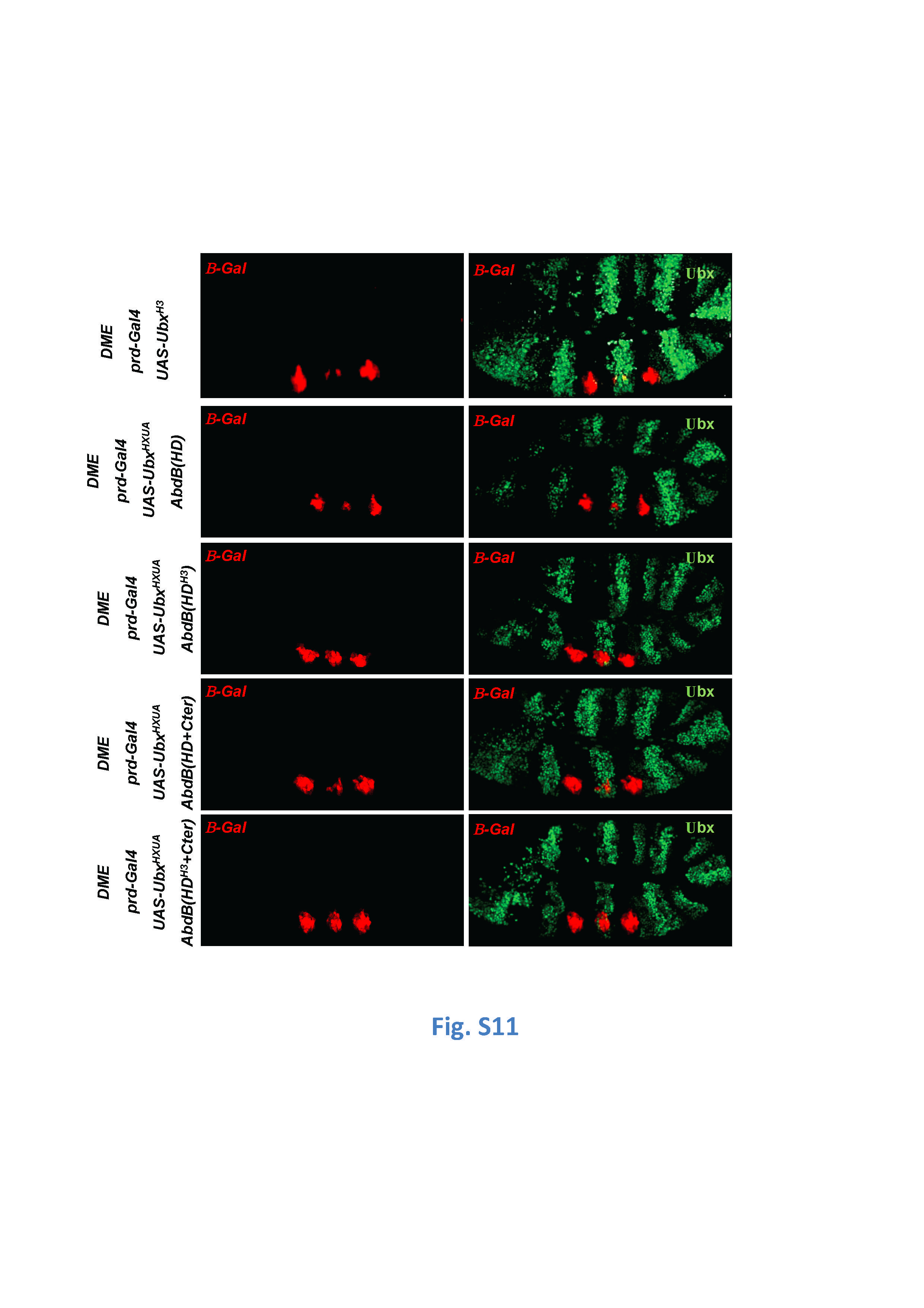

Supplement: Figure S11 — Ubx/AbdB chimera protein sequence requirements for DME repression. Thoracic centered magnifications of embryo bearing the DME reporter co-stained for β-gal (red) and UbxH3 or Ubx/AbdB chimeras (green) driven by prd-Gal4. Levels of UbxH3 or Ubx/AbdB chimeras repressive activity on DME was evaluated by defining the ratio of β-gal staining in T2 and T3 (100% of repressive activity was given when the T2/T3 ratio was 1, and 0% when the T2/T3 ratio was O). Illustrations for wild type AbdB is given in Figure 1J, 1K, and for UbxHX,UA in [15]. Quantifications are shown in Figure 7. (TIF) [file pgen.1003307.s011.tif]
